# Supplementary material for: Host, parasite and drug determinants of clinical outcomes following treatment of visceral leishmaniasis: a protocol for individual participant data meta-analysis
Source: BMJ Open. 2023 Oct 28;13(10):e074679. doi: 10.1136/bmjopen-2023-074679 (PMC10618999; doi:10.1136/bmjopen-2023-074679)
Supplement: Supplementary data [file bmjopen-2023-074679supp001.pdf]

Example of Search Strategies: The dates will be different for future searches

Pubmed

((((((((randomized controlled trial[pt] OR controlled clinical trial[pt] OR randomized[tiab] OR placebo[tiab] OR clinical trials as topic[mesh:noexp] OR randomly[tiab] OR trial[ti] NOT (animals[mh] NOT humans [mh]))) AND Clinical Trial[ptyp])) AND (((("Leishmaniasis, Visceral"[Mesh]) OR visceral leishmaniasis[Title/Abstract]) OR kala azar[Title/Abstract]) OR black fever[Title/Abstract]))) OR (((((((((((((("Pentamidine"[Mesh]) OR "liposomal amphotericin B" [Supplementary Concept]) OR "Amphotericin B"[Mesh]) OR "Paromomycin"[Mesh]) OR "miltefosine" [Supplementary Concept]) OR "Sodium"[Mesh]) OR "8-aminoquinoline" [Supplementary Concept]) OR "Ketoconazole"[Mesh]) OR "Azoles"[Mesh]) OR "Allopurinol"[Mesh]) OR ("Atovaquone"[Mesh] OR "atovaquone, proguanil drug combination" [Supplementary Concept]))) OR ((pentamidine[Title/Abstract] OR ambisome[Title/Abstract] OR amphotericin[Title/Abstract] OR paromomycin[Title/Abstract] OR miltefosine[Title/Abstract] OR pentavalent[Title/Abstract] OR sodium[Title/Abstract] OR sitamaquine[Title/Abstract] OR azole*[Title/Abstract] OR allopurinol[Title/Abstract] OR atovaquone[Title/Abstract] OR ketoconazole[Title/Abstract] OR fluconazole[Title/Abstract] OR metronidazole[Title/Abstract])))) AND (((("Leishmaniasis, Visceral"[Mesh]) OR visceral leishmaniasis[Title/Abstract]) OR kala azar[Title/Abstract]) OR black fever[Title/Abstract])))) NOT (((animals not humans)))
Filters activated: Publication date from 2016/01/01 to 2020/12/31.

- This search strategy uses the PubMed RCT filter for sensitivity- and precision-maximising version (2008). https://work.cochrane.org/pubmed

Database: Embase 1974 to present

Search Strategy:

----

1 exp visceral leishmaniasis/ (9675)
2 "black fever".ti,ab. (17)
3 "kala azar".ti,ab. (2227)
4 "visceral leishmaniasis".ti,ab. (9360)
5 1 or 2 or 3 or 4 (12383)
6 exp randomized controlled trial/ (603493)
7 Controlled clinical study/ (464274)
8 Random$.ti,ab. (1531396)
9 randomization/ (86779)
10 intermethod comparison/ (260073)
11 placebo.ti,ab. (305154)
12 (compare or compared or comparison).ti. (507638)
13 ((evaluated or evaluate or evaluating or assessed or assess) and (compare or compared or comparing or comparison)).ab. (2100417)
14 (open adj label).ti,ab. (78868)
15 ((double or single or doubly or singly) adj (blind or blinded or blindly)).ti,ab. (231216)
16 double blind procedure/ (172220)
17 parallel group$1.ti,ab. (25399)
18 (crossover or cross over).ti,ab. (104779)

19 ((assign$ or match or matched or allocation) adj5 (alternate or group$1 or intervention$1 or patient$1 or subject$1 or participant$1)).ti,ab. (328145)
20 (assigned or allocated).ti,ab. (386517)
21 (controlled adj7 (study or design or trial)).ti,ab. (346624)
22 (volunteer or volunteers).ti,ab. (245818)
23 trial.ti. (298535)
24 6 or 7 or 8 or 9 or 10 or 11 or 12 or 13 or 14 or 15 or 16 or 17 or 18 or 19 or 20 or 21 or 22 or 23 (4650673)
25 5 and 24 (1506)
26 (pentamidine or ambisome or amphotericin or paromomycin or miltefosine or pentavalent or sodium or sitamaquine or azole* or allopurinol or atovaquone or ketoconazole or fluconazole or metronidazole).mp. (1012481)
27 5 and 26 (3701)
28 25 or 27 (4786)
29 (exp animal/ or animal.hw. or nonhuman/) not (exp human/ or human cell/ or (human or humans).ti.) (6396469)
30 28 not 29 (3730)
31 30 (3730)
32 limit 31 to yr="2016 -Current" (774)

- This search strategy uses the Cochrane RCT filter for Embase. https://www.cochranelibrary.com/central/central-creation

Scopus

((TITLE-ABS-KEY ("visceral leishmaniasis" OR "kala azar" OR "black fever")) AND ((TITLE-ABS-KEY (random* OR rct OR placebo OR allocat* OR crossover* OR "cross over" OR trial OR (doubl* W/1 blind*) OR (singl* W/1 blind*))) OR (TITLE-ABS-KEY (control* W/1 trial*)) OR (TITLE-ABS-KEY (cohort*)))) OR ((TITLE-ABS-KEY ("visceral leishmaniasis" OR "kala azar" OR "black fever")) AND (TITLE-ABS-KEY (pentamidine OR ambisome OR amphotericin OR paromomycin OR miltefosine OR pentavalent OR sodium OR sitamaquine OR azole* OR allopurinol OR atovaquone OR ketoconazole OR fluconazole OR metronidazole))) AND (EXCLUDE (EXACTKEYWORD , "Animals") OR EXCLUDE (EXACTKEYWORD , "Animal") OR EXCLUDE (EXACTKEYWORD , "Animal Experiment") OR EXCLUDE (EXACTKEYWORD , "Mouse") OR EXCLUDE (EXACTKEYWORD , "Mice") OR EXCLUDE (EXACTKEYWORD , "Dogs")) AND (LIMIT-TO (LIMIT-TO (PUBYEAR , 2020) OR (PUBYEAR , 2019) OR LIMIT-TO (PUBYEAR , 2018) OR LIMIT-TO (PUBYEAR , 2017) OR LIMIT-TO (PUBYEAR , 2016))

Web of Science Core Collection

1. TOPIC: ("visceral leishmaniasis" OR "kala azar" OR "black fever")
2. TOPIC: (random* OR rct OR placebo OR allocat* OR crossover* OR "cross over" OR trial OR (doubl* near/1 blind*) OR (singl* near/1 blind*))
3. TOPIC: (control* near/1 trial*)
4. TOPIC: (cohort*)
5. #4 OR #3 OR #2
6. #5 AND #1
7. TOPIC: (pentamidine OR ambisome OR amphotericin OR paromomycin OR miltefosine OR pentavalent OR sodium OR sitamaquine OR azole* OR allopurinol OR atovaquone OR ketoconazole OR fluconazole OR metronidazole)

8. #7 AND #1
9. #8 OR #6
10. #8 OR #6
11. Refined by: PUBLICATION YEARS: (2020 OR 2019 OR 2018 OR 2017 OR 2016)

Cochrane Central Register of Controlled Trials
Issue 5 of 12, May 2020

#1	MeSH descriptor: [Leishmaniasis, Visceral] explode all trees	39
#2	"black fever"	1
#3	"kala azar"	119
#4	"visceral leishmaniasis"	242
#5	#1 or #2 or #3 or #4	283

Custom Range: 2016 to 2020

World Health Organization Global Index Medicus https://www.globalindexmedicus.net/

tw:(tw:(("visceral leishmaniasis" OR "kala azar" OR "black fever")) AND (instance:"ghl") AND (year_cluster:("2016" OR "2017" OR "2018" OR "2019" OR “2020”))) AND (instance:"ghl")

ClinicalTrials.gov Advanced Search https://clinicaltrials.gov/ct2/search/advanced?

Condition or disease: visceral leishmaniasis OR kala azar OR black fever

WHO International Clinical Trials Registry Platform http://apps.who.int/trialsearch/

visceral leishmaniasis OR kala azar OR black fever – Trials at ALL stages
2016 – date

NOTE: THIS PLATFORM IS UNAVAILABLE ON 14/05/2020
