# Supplementary material for: Host, parasite and drug determinants of clinical outcomes following treatment of visceral leishmaniasis: a protocol for individual participant data meta-analysis
Source: BMJ Open. 2023 Oct 28;13(10):e074679. doi: 10.1136/bmjopen-2023-074679 (PMC10618999; doi:10.1136/bmjopen-2023-074679)
Supplement: Author's manuscript [file bmjopen-2023-074679.draft_revisions.pdf]

BMJ Open

BMJ Open is committed to open peer review. As part of this commitment we make the peer review history of every article we publish publicly available.

When an article is published we post the peer reviewers' comments and the authors' responses online. We also post the versions of the paper that were used during peer review. These are the versions that the peer review comments apply to.

The versions of the paper that follow are the versions that were submitted during the peer review process. They are not the versions of record or the final published versions. They should not be cited or distributed as the published version of this manuscript.

BMJ Open is an open access journal and the full, final, typeset and author-corrected version of record of the manuscript is available on our site with no access controls, subscription charges or pay-per-view fees (http://bmjopen.bmj.com).

If you have any questions on BMJ Open's open peer review process please email info.bmjopen@bmj.com

BMJ Open

Host, parasite, and drug determinants of clinical outcomes following treatment of visceral leishmaniasis: a protocol for individual participant data meta-analysis

Journal:	*BMJ Open*
Manuscript ID	bmjopen-2023-074679
Article Type:	Protocol
Date Submitted by the Author:	13-Apr-2023
Complete List of Authors:	Kumar, Rishikesh; Rajendra Memorial Research Institute of Medical Sciences (RMRIMS)<br>Dahal, Prabin; Infectious Diseases Data Observatory (IDDO); University of Oxford, Centre for Tropical Medicine and Global Health, Nuffield Department of Clinical Medicine<br>Singh-Phulgenda, Sauman; Infectious Diseases Data Observatory (IDDO); University of Oxford, Centre for Tropical Medicine and Global Health, Nuffield Department of Clinical Medicine<br>Siddiqui, Niyamat; Rajendra Memorial Research Institute of Medical Sciences (RMRIMS)<br>Munir, Abdalla; Infectious Diseases Data Observatory; University of Oxford Centre for Tropical Medicine and Global Health, Centre for Tropical Medicine and Global Health, Nuffield Department of Clinical Medicine<br>Naylor, Caitlin; Infectious Diseases Data Observatory (IDDO); University of Oxford, Centre for Tropical Medicine and Global Health, Nuffield Department of Clinical Medicine<br>Wilson, James; Infectious Diseases Data Observatory (IDDO); University of Oxford, Centre for Tropical Medicine and Global Health, Nuffield Department of Clinical Medicine<br>Buck, Gemma; Infectious Diseases Data Observatory (IDDO); University of Oxford, Centre for Tropical Medicine and Global Health, Nuffield Department of Clinical Medicine<br>Rahi, Manju; ICMR, Epidemiology and Communicable Diseases<br>Malaviya, Paritosh; Banaras Hindu University, Department of Medicine, Institute of Medical Sciences<br>Alves, Fabiana; Drugs for Neglected Disease Initiative<br>Sundar, Shyam; Banaras Hindu Univ<br>Ritmeijer, Koert; Medecins Sans Frontieres, Public Health Department<br>Stepniewska, Kasia; Infectious Diseases Data Observatory (IDDO); University of Oxford, Centre for Tropical Medicine and Global Health, Nuffield Department of Clinical Medicine<br>Guérin, Philippe J.; Infectious Diseases Data Observatory/University of Oxford<br>Pandey, Krishna; Rajendra Memorial Research Institute of Medical Sciences (RMRIMS)
Keywords:	EPIDEMIOLOGY, Neglected Diseases, INFECTIOUS DISEASES, PUBLIC HEALTH, Tropical medicine < INFECTIOUS DISEASES

SCHOLARONE™
Manuscripts

Title: Host, parasite, and drug determinants of clinical outcomes following treatment of visceral leishmaniasis: a protocol for individual participant data meta-analysis

Rishikesh Kumar [1], Prabin Dahal [2,3§], Sauman Singh-Phulgenda [2,3], Niyamat Ali Siddiqui [1], Abdalla Munir [2,3], Caitlin Naylor [2,3], James Wilson[2,3], Gemma Buck [2,3], Manju Rahi [4], Paritosh Malaviya [5], Fabiana Alves [6], Shyam Sundar [5], Koert Ritmeijer[7], Kasia Stepniewska [2,3], Philippe J Guerin[2,3], Krishna Pandey [1§]

[1] ICMR – Rajendra Memorial Research Institute of Medical Sciences (RMRIMS), Patna, India

[2]Infectious Diseases Data Observatory (IDDO), Oxford, UK

[3]Centre for Tropical Medicine and Global Health, Nuffield Department of Clinical Medicine, University of Oxford, Oxford, UK

[4]Indian Council of Medical Research (ICMR), New Delhi, India

[5]Infectious Disease Research Laboratory, Department of Medicine, Institute of Medical Sciences, Banaras Hindu University, Varanasi, India

[6]Drugs for Neglected Diseases initiative, Geneva, Switzerland

[7]Médecins Sans Frontières, Amsterdam, Netherlands

[§]*Correspondence: drkrishnapandey@gmail.com, prabin.dahal@ndm.ox.ac.uk*

For peer review only - http://bmjopen.bmj.com/site/about/guidelines.xhtml

For peer review only

Abstract

Introduction: Visceral leishmaniasis (VL) is a parasitic disease with an estimated 50,000 to 90,000 new cases occurring annually. There is an observed variation in the efficacy of the current first line therapies across different regions. Such heterogeneity could be a function of host, parasite, and drug factors. An individual participant data meta-analysis (IPD-MA) is planned to explore the determinants of treatment outcomes.

Methods and analysis: The Infectious Diseases Data Observatory (IDDO) VL living systematic review (IDDO VL LSR) library is an open-access resource of all published therapeutic studies in VL since 1980 (PROSPERO registration: CRD42021284622). Studies indexed in the IDDO VL LSR library were screened for eligibility for inclusion in this IPD-MA. Corresponding authors and principal investigators of the studies meeting the eligibility criteria for inclusion were invited to be part of the collaborative IPD-MA. Authors agreeing to participate in this collaborative research were requested to share the IPD using the IDDO (IDDO) VL data platform. The IDDO VL data platform currently holds datasets from clinical trials standardised to a common data format and provides a unique opportunity to identify host, parasite, and drug determinants of treatment outcomes. Multivariable regression models will be constructed to identify determinants of therapeutic outcomes using generalised linear mixed effects models accounting for within-study site clustering.

Ethics and dissemination: The results of this IPD-MA will be disseminated at conferences, IDDO website and any peer-reviewed publications. All publications will be open source. Findings of this research will be critically important for the control programmes at regional/global levels, policy makers and groups developing new VL treatments.

For peer review only

For peer review only - http://bmjopen.bmj.com/site/about/guidelines.xhtml

PROSPERO registration: The systematic review component of the IPD-MA has the following PROSPERO registration: CRD42021284622.

Author Email:

RK drkumarrishikesh@gmail.com
PD prabin.dahal@iddo.org
SSP sauman.singh@iddo.org
NAS niyamatalisiddiqui@yahoo.com
AM abdalla.munir@iddo.org
CN caitlin.naylor@iddo.org
JW james.wilson@iddo.org
GB gemma.buck@iddo.org
MR drmanjurahi@gmail.com
PM paritosh_malaviya@yahoo.com
FA falves@dndi.org
SS drshyamsundar@hotmail.com
KR koert.ritmeijer@amsterdam.msf.org
KS kasia.stepniewska@iddo.org
PJG philippe.guerin@iddo.org
KP drkrishnapandey@yahoo.com

For peer review only

Keywords

Visceral Leishmaniasis; Therapeutic Outcome; Statistical Analysis Plan, meta-analysis, individual participant data; relapse

Word count: 3478 (main text); abstract (255)

For peer review only - http://bmjopen.bmj.com/site/about/guidelines.xhtml

Strengths and limitations of this study

- In any single Visceral Leishmaniasis clinical trial, only few relapses are observed which limits the ability to identify predictors associated with it. An individual participant data (IPD-MA) will increase the statistical power to detect the predictors and moderators of treatment outcome.

- The identification of studies eligible for inclusion in the IPD-MA has been made through a comprehensive literature search of all published studies since 1980 with pre-defined inclusion-exclusion criteria. However, retrieval of data from trials published prior to 2000 can be a major challenge.

- This IPD-MA will utilise the VL repository of individual participant data hosted by Infectious Diseases Data Observatory (IDDO VL data platform). A major strength of this study is that data on the IDDO VL data platform is harmonised to a common standard based on an extensive consultation with the VL research community.

- A particular scientific challenge is that distinction of relapse from re-infection is seldom carried out in VL therapeutic trials and hence won't be considered in this analysis.

For peer review only

For peer review only - http://bmjopen.bmj.com/site/about/guidelines.xhtml

Introduction

Visceral leishmaniasis (VL), also known as kala-azar, is a parasitic disease with anthroponotic and zoonotic modes of transmission [1]. The disease is characterised by prolonged fever, cachexia, splenomegaly, hepatomegaly and anaemia. The annual global incidence of VL is estimated at 50,000 to 90,000 cases, with only 25-45% of the cases reported to the World Health Organization [2]. The disease has an outbreak potential and linked with war, conflicts and climate change, and if left untreated, the disease is fatal over 95% of the cases [2–4].

The efficacy of antileishmanial regimens in VL varies across geographical regions. For example, single dose liposomal amphotericin B (L-AmB) has a demonstrated high efficacy (≥94%) in the Indian sub-continent while its efficacy is sub-optimal (58%) in East Africa [5,6]. There are high levels of resistance against antimony-based drugs in India, although it continues to be used in a combination regimen with paromomycin as a first line therapy in East Africa [5,6]. The underlying reasons for such variation are not fully understood but the observed therapeutic response is likely multifactorial and may be a function of drug resistance, variation in the pharmacokinetic and pharmacodynamic properties of the drugs used and the underlying host immunity [7–10].

Several studies have reported on the determinants of therapeutic outcomes in studies conducted across different geographical regions and patient populations [11–16]. Additionally, the heterogeneity introduced by variation in study design and conduct leads to challenges in reliably comparing the effect measures across the studies. A major challenge in reliably assessing risk factors of therapeutic outcomes is that at individual study level, the number of events is often small, which limits the precision of the estimated effect.

For peer review only

For peer review only - http://bmjopen.bmj.com/site/about/guidelines.xhtml

116 Individual participant data (IPD) meta-analysis (IPD-MA) of existing studies can overcome this limitation by increasing the effective sample size and allows for greater power in detecting differential treatment effects across individuals in trials [17].

Objectives

The objectives of this IPD-MA are:

- To identify host, parasite, and drug determinants of initial cure after the completion of treatment schedule
- To identify host, parasite, and drug determinants of definitive cure at 6 months after treatment completion
- To identify host, parasite, and drug determinants of relapse at 6 months after treatment completion
- To identify host, parasite, and drug determinants of mortality outcome at any time point during the study

Primary and secondary endpoints relating to these objectives are defined in the outcomes section below.

For peer review only - http://bmjopen.bmj.com/site/about/guidelines.xhtml

Methods and analysis: Patients, interventions and outcomes

PICOT statement

Population: Any patient enrolled in an interventional study with a confirmed or suspected diagnosis of VL defined by serological and/or parasitological testing.

Interventions: Any antileishmanial therapy

Comparator: Not restricted by the use of a comparator drug

Outcome: At least one of the following outcomes reported: initial cure, definitive cure, relapse, or fatality

Time: Studies published on or after 1980

Criteria for study eligibility

The following eligibility criteria must be fulfilled for study inclusion:

- Prospective clinical efficacy studies on patients with confirmed or suspected VL either using microscopy/serology/molecular method (i.e. clinical diagnosis followed by a confirmatory method)
- Information on constituent drug(s), dose and duration of treatment regimen is available
- At least one of the following clinical outcomes are measured: initial cure, definitive cure, relapse or mortality

For peer review only - http://bmjopen.bmj.com/site/about/guidelines.xhtml

For peer review only

Criteria for patient eligibility

The following minimum information are required for inclusion of a patient in the IPD meta-analysis:

- Details of antileishmanial treatment(s) administered
- Baseline information on age and gender
- Outcome is recorded

Outcomes

Outcomes and definitions:

Primary endpoint

The primary endpoint adopted is cure at 6-months following treatment completion. This is commonly referred to as 'definitive' or 'final' cure. The precise definition and timing of definitive cure will vary according to study design. As a minimum, it is expected that a definitive cure is achieved if the patient remains alive and is no longer be manifesting symptoms and/or signs of visceral leishmaniasis.

Further analysis of this endpoint will be carried out using a stricter definition of definitive cure, defined as: achievement of initial cure (as defined below) and no subsequent rescue treatment or evidence of clinical or parasitological relapse, and no death associated with VL during the 6-months following treatment completion.

Secondary endpoints:

The following secondary endpoints are adopted:

For peer review only

For peer review only - http://bmjopen.bmj.com/site/about/guidelines.xhtml

Initial cure: Initial cure is defined at the time of initial assessment adopted in the original study after completion of the therapy along with clinical improvement. The timing of initial cure assessment would typically take place within 28 days of treatment completion but this varies slightly across studies.

Further analysis of this endpoint will be carried out using a stricter definition of initial cure, defined as: cure at day 28 or at time-point defined in the original study following treatment, defined as clinical improvement of VL, absence of parasites in the spleen or bone marrow using microscopy) and no rescue therapy on or before day 28 or before the timepoint of initial cure assessment adopted in the original study.

Relapse: Relapse is defined as recurrence of signs and symptoms of VL at any time point during the study follow-up among those who achieved initial cure. The assessment typically takes place at 6 months but this varies slightly across studies.

Tissue aspirates for confirming parasitological presence are usually carried out based on clinical suspicion of relapse during the follow-up [18,19]. Since tissue aspiration for defining relapse won't be carried out in all the studies, further analysis of this endpoint will be restricted to studies that defined relapse based on parasitological demonstration.

Mortality: This is defined as deaths reported as related to the study interventions or due to the progressive worsening of the disease itself at any time during the study. Further analysis of this endpoint will be carried out by considering all-cause mortality.

For peer review only

For peer review only - http://bmjopen.bmj.com/site/about/guidelines.xhtml

Variation is time-points:

The assessment of initial cure and definitive cure is usually undertaken at 1-month and 6-months post treatment. However, the exact time of these assessment will vary across the studies. Assessments undertaken between 15 to 60 days will be considered as time of initial cure. For assessments of definitive cure and relapse, assessments made between 5 to 7 months will be considered as 6 months.

Statistical methods

Identification of relevant studies

Eligible studies were identified from the infectious diseases data observatory (IDDO) library of all prospective therapeutic studies that has systematically indexes all published therapeutic efficacy studies in VL published from 1980 onwards [20,21]. The IDDO VL clinical trials library is based on a living systematic review and the database is continually updated every six months in accordance with the Preferred Reporting Items for Systematic-Reviews and Meta-Analyses (PRISMA) guidelines [22]. The trial library indexes publications identified from the following databases: PubMed, Embase, Scopus, Web of Science, Cochrane, clinicaltrials.gov, WHO ICTRP, Global Index Medicus, IMEMR, IMSEAR, and LILACS. For this current review, the search includes all clinical trials published between 1st of Jan 1980 and 2nd of May 2021. Details of the search strategy adopted is described elsewhere [22]. Studies indexed in the IDDO VL library were eligible for inclusion in this review if they meet the inclusion and exclusion criteria outlined above. This review is not limited by language.

For peer review only - http://bmjopen.bmj.com/site/about/guidelines.xhtml

Collation of IPD

Authors (principal investigators, corresponding authors) of the studies in the IDDO VL LSR library eligible for the inclusion in this IPD-MA were invited to be part of this collaborative research. Researchers agreeing to the terms and conditions of the submission were then requested to upload IPD to the IDDO VL data platform through a secured web portal [23]. Data in the IDDO VL platform are fully pseudonymised to protect personal information and patient privacy.

Data management

Raw data from individual studies shared with IDDO VL data platform are currently being standardised using the Clinical Data Interchange Standards Consortium (CDISC) compliant curation standards [24]. If required, investigators will be further contacted to clarify questions that arise during data curation and analysis, and individual study protocols will be requested. On standardisation, the data are stored in a relational database containing information on drug regimen, parasitological, clinical, and haematological assessments, and therapeutic outcomes.

For peer review only - http://bmjopen.bmj.com/site/about/guidelines.xhtml

Statistical methods for primary and secondary outcomes

Variables considered for regression modelling: The following variables will be considered for inclusion in the analysis of primary and secondary endpoints:

Host variables: The following host variables are considered: age, gender, body weight, nutritional status, comorbidity status (such as HIV), and duration of illness prior to study enrolment. The following baseline clinical measurements will be considered: haemoglobin/anaemia status, spleen size, immunological biomarkers (CD4+ count), alanine transaminase (ALT) and aspartate transaminase (AST), platelets and neutrophils. If data are available, the following baseline characteristics will be considered: history of blood transfusion, bilirubin, creatinine, urea, and albumin concentrations.

Parasite variables: The following parasite related baseline factors will be considered: parasite load and information regarding the nature of infection (primary vs. previously treated cases). Any cases described as previously untreated (or "fresh") cases for leishmaniasis will be considered as primary VL. The enumeration of parasite density is usually carried out by evaluating bone marrow or splenic aspirates or through quantitation of parasites in unit quantity of specimen or parasite DNA equivalence per PCR reaction across studies[25–28]; the Chulay-Bryceson scale being the most commonly adopted [25]. The methodology used for parasite gradation will be considered in the analysis.

Drug variables: The following drug related variables will be considered for inclusion in analysis: mg/kg total dose (or target dose) administered [29], treatment duration, whether the regimen was monotherapy or a combination therapy, mode of drug administration (intravenous, intramuscular or oral), administration of regimen either as a single dose or as a multiple doses, direct or indirect observations of drug administration for oral medications.

For peer review only - http://bmjopen.bmj.com/site/about/guidelines.xhtml

The dosage of antileishmanial interventions will be determined and expressed as dose per unit body weight (e.g., total mg/kg dosage administered) and will be considered separately for each drug regimens.

Study level variables: The following study or arm level variables will be considered in the analysis of primary and secondary endpoints: geographical region, country, study site, and calendar year of the study conduct.

Statistical analysis and reporting

Descriptive summary:

A summary of included studies will be presented with respect to study location, year of study conduct, characteristics of study population, duration of follow-up, details of drug regimens including supervision of drug administration, and methodological details adopted for disease confirmation and treatment outcomes.

Summary of baseline characteristics of the patients included in the analysis will be presented by geographical region and overall. These include: age; weight; parasite grade at enrolment; temperature; haemoglobin (or haematocrit) concentration; anaemia and severe anaemia status, spleen size, description of severity of infection (severe /mild/uncomplicated) if available, total mg/kg dose for each treatment, and supervision of drug administration. The distribution of the baseline characteristics will be summarised either as a proportion for categorical variables, as the mean (with standard deviation) or median (with interquartile range) for continuous variables.

For peer review only - http://bmjopen.bmj.com/site/about/guidelines.xhtml

For peer review only

Analysis of the primary endpoint:

A mixed effects logistic regression model will be used for identifying the risk factors for definitive cure in a one-stage IPD-MA. Given a relatively low number of expected events in each included study, to avoid imprecise estimates from small events and continuity corrections, a one-stage IPD approach is preferred [30]. A random intercept regression model will be considered with study sites specified as random effects to account for within-site clustering of the patients (as some of the trials are multicentre studies).

If information regarding the time of occurrence of event is available, Kaplan-Meier (K-M) method will be used to estimate the probability of definitive cure at the end of the study follow-up, and a shared frailty Cox regression model will be considered with study site fitted as a frailty term to account for between-study site heterogeneity. The following outcomes will be censored in the survival analysis: lost to follow-up, death due to causes adjudicated as unrelated to the study drug, voluntary withdrawal from the study.

Multivariable modelling

Core predictor set

The following set of variables are the risk factors reported in clinical literature for treatment outcomes in VL: age, sex, baseline parasite density, HIV co-infection, and geographical region. These variables along with the drug regimen will form the minimal adjustment set for assessment of other risk factors and will be kept in the regression model regardless of statistical significance [31].

For peer review only - http://bmjopen.bmj.com/site/about/guidelines.xhtml

Assessment of other predictors and considerations for multivariable modelling

The association between the remaining candidate predictors and therapeutic outcomes will be assessed by adjusting for the core predictor sets identified. Multivariable model construction will follow the recommendations of Heinze et al (2017) [32]. Nested models will be compared by assessing the change in log-likelihood estimates and Akaike's information criterion (AIC) will be used for comparing competing non-nested models. The functional form of the continuous variables will be determined using multivariable fractional polynomials [33] or restricted cubic splines [31]. Stability investigations will be undertaken to account for uncertainty introduced in multivariable modelling through bootstrap resampling [32].

Missing data

The proportion of missing observation for each of the variables considered in the analysis will be summarised and any missingness patterns will be explored. Multiple imputation [34], which assumes missing at random mechanism for missingness, will be undertaken to handle missing observations. Construction of the imputation model will include all the variables in the target analysis (i.e., all included exposures, outcome in the target analysis), and additional auxiliary variables including any interaction terms and nonlinear associations. The number of imputations will be determined based on the fraction of missing information. The target analysis will then be carried out in each of the completed (observed plus imputed values) datasets and the estimates will be combined across the imputed datasets using Rubin's combination rules [34].

For peer review only - http://bmjopen.bmj.com/site/about/guidelines.xhtml

Subgroup analyses

The following two analyses are planned:

- There is a known regional variation in treatment response in VL [5]. Therefore, interaction between treatment and geographical region will be investigated. A separate analysis could be undertaken for each of the geographical regions.
- Patients living with HIV who are treated for VL typically have worse outcomes and higher mortality risk than those who are not living with HIV [20,35]. Although, usually an exclusion criterion of therapeutic trials, a separate sub-group analysis will be carried out among patients with defined VL-HIV coinfections (data permitting). An interaction between treatment and HIV status (treatment-covariate interaction) will therefore be considered; within-study interaction will be separated from the between-study interaction by centering the covariates [36].

Sensitivity analyses

Stability investigations will be undertaken to account for uncertainty introduced in multivariable modelling through bootstrap resampling. The robustness of the derived estimates and their variance will be summarised using the recommendations in Heinze et al (2017) [32]. The influence of each of the studies towards the estimated regression coefficients will be assessed by removing each study at a time and estimating the coefficient of variation for the parameter estimates.

For peer review only - http://bmjopen.bmj.com/site/about/guidelines.xhtml

Analysis of secondary endpoints

Initial cure: Proportion of patients achieving initial cure (as defined previously) in each of the studies will be presented. The construction of univariable and multivariable regression models will follow the same approach as for the primary endpoint.

Relapse: Proportion of patients achieving relapse (as defined previously) in each of the studies will be presented. The construction of univariable and multivariable regression models will follow the same approach as for the primary endpoint.

Mortality: Proportion of deaths reported in each of the studies will be presented. The construction of univariable and multivariable regression models will follow the same approach as for the primary endpoint.

Software

All analyses will be carried out using R software or Stata 17 software [37,38]. Use of any other data analysis tools will not change the statistical analysis plan.

Risk of bias assessment in included studies and in the IPD-MA

To examine the risk of bias in included studies, the Cochrane risk of bias tool (RoB 2) for randomised studies [39]. Risk of bias in non-randomised studies will be carried out by assessing the pre-intervention, at intervention and post-intervention domains as outlined in using ROBINS-I tool [40].

To examine the risk of bias in IPD-MA, the first four domains of the quality In prognosis studies (QUIPS) tool and the first three domains of the prediction model risk of

For peer review only - http://bmjopen.bmj.com/site/about/guidelines.xhtml

bias assessment tool (PROBAST) will be considered as recommended in Riley et al (2021) [31]. The relevant domains from the QUIPS checklist are study participation, study attrition, prognostic factor measurement, and outcome measurement, and the relevant domains from PROBAST checklist are patient selection, prognostic factors, and outcomes. Two reviewers will independently assess the risk of bias in the studies included in the analysis.

Assessment of risk of potential bias in missing studies

Despite best possible efforts, it is anticipated that raw data from all the identified studies will not be available. The characteristics of patient population and study meta-data from the missing studies will be summarised to explore if the missing studies are systematically different from the studies that are included in the meta-analysis. A two-stage IPD may be conducted if sufficient details (or any covariate adjustment) are reported in the original studies.

For peer review only

For peer review only - http://bmjopen.bmj.com/site/about/guidelines.xhtml

Dissemination plans

Ethics and dissemination

This IPD-MA meets the criteria for waiver of ethical review as defined by the Oxford Tropical Research Ethics Committee (OxTREC) granted to IDDO, as the research consists of secondary analysis of existing anonymised data. Ethical approval was granted to each study included in this pooled analysis by their respective ethics committees. This IPD-MA will address research questions similar to that of included studies. Findings of this IPD-MA will be reported in open-access, peer-reviewed journals following the PRISMA-IPD guidelines [41].

Patient and public involvement

The research questions considered in this IPD-MA is based on a research agenda developed by the global VL research community [42]. The design and development of this IPD-MA were done by the study authors only and no patient was involved at any stage.

Further development of statistical analysis plan

Major statistical analyses have been included in this plan. Amendments to the current plan or additional statistical analyses may be required as data accrual is in progress.

For peer review only - http://bmjopen.bmj.com/site/about/guidelines.xhtml

Discussion

Global commitment over the past few decades has led to significant progress in the control and elimination of VL. In the Indian subcontinent (ISC) the VL burden has greatly reduced in the past 15 years [43,44]. Availability of effective antileishmanial treatment is the cornerstone for achieving and sustaining elimination in the ISC. As the overall incidence of VL is falling, a growing proportion of the total reported cases in the region is attributed to relapses [44]. Similarly, a recent report assessing long-term epidemiological trend in South Sudan has also indicated increasing incidence of relapses over the past two decades [45]. This is an important public health concern as patients with relapse are predisposed to further relapse, especially in those with HIV co-infections, [12] and thus can fuel the onwards transmission the disease. Therefore, identification of host, parasite or drug related characteristics remain crucial for effective case management.

Several clinical trials and meta-analyses have highlighted the therapeutic efficacy and safety of one or more antileishmanial therapies [5,11–13,16,46]. However, risk factors determining the undesirable therapeutic outcomes remain poorly understood. Underlying heterogeneities in the published studies in terms of study population, treatment regimen, outcome definitions, and study designs render difficulties in drawing a comprehensive conclusion regarding drug efficacy. Some of these difficulties can be ameliorated through an IPD-MA [17]. We hope that this IPD-MA will provide evidence for the efficacy of different treatments in VL that can be further considered by policy makers at regional and global levels.

For peer review only - http://bmjopen.bmj.com/site/about/guidelines.xhtml

The identification of studies eligible for inclusion in the IPD-MA has been made through a comprehensive literature search of all published studies since 1980 with pre-defined inclusion-exclusion criteria. A major strength of this study is that data from several studies will be harmonised to a common standard based on an extensive consultation with the VL research community [24], thus allowing us to address some of the methodological sources of heterogeneity.

A major challenge is that a substantial proportion of the studies in the IDDO library were conducted prior to the year 2000; the retrieval of data from historical trials is a major challenge. A particular scientific challenge is that misclassification of re-infection as "true" relapse can potentially introduce bias in relapse estimates. A distinction of relapse and re-infection is seldom carried out in VL therapeutic trials and hence won't be considered in this analysis. Evidence form a study conducted in Nepal that used parasite genotyping suggested late relapses were less likely to be new infections [18], but generalisability to other settings remains unclear. Finally, the determinants of therapeutic outcomes in this IPD-MA would be limited to the commonly assessed and available clinical-laboratory parameters across VL drug trials.

Conclusion

This IPD-MA will combine IPD from therapeutic studies from the IDDO VL data platform to identify the determinants of therapeutic outcomes. Findings of this research will generate important information for the control programmes at regional and global levels, policy makers and groups developing new VL treatments.

For peer review only - http://bmjopen.bmj.com/site/about/guidelines.xhtml

Authors' contributions

All authors listed were responsible for the study conceptualisation. RK, PD, KP, PJG, KS wrote the first draft of the manuscript. All authors were involved in reading, revising it critically, editing and approving the final manuscript.

Funding

This work is funded by a Bill & Melinda Gates Foundation grant to the Infectious Diseases Data Observatory, Oxford University, UK (Recipient: Prof. Philippe Guerin; ref: INV-004713).

Conflict of Interest

None

Data Availability

N/A as this is an analysis protocol.

For peer review only

For peer review only - http://bmjopen.bmj.com/site/about/guidelines.xhtml

References

1. Boelaert M, Sundar S. Leishmaniasis. In: Farrar J, Hotez PJ, Junghanss T, Kang G, Lalloo D, White NJ, editors. Manson's Tropical Infectious Diseases, Twentry-third edition. Elsevier Health Sciences; 2014. pp. 631-651.e4. doi:https://doi.org/10.1016/B978-0-7020-5101-2.00048-0
2. World Health Organization. Leishmaniasis: Key facts. In: WHO [Internet]. 2023 [cited 27 Feb 2023]. Available: https://www.who.int/news-room/fact-sheets/detail/leishmaniasis
3. Collin S, Davidson R, Ritmeijer K, Keus K, Melaku Y, Kipngetich S, et al. Conflict and kala-azar: Determinants of adverse outcomes of kala-azar among patients in southern Sudan. Clin Infect Dis. 2004;38: 612–619. doi:10.1086/381203
4. Al-Salem W, Herricks JR, Hotez PJ. A review of visceral leishmaniasis during the conflict in South Sudan and the consequences for East African countries. Parasites and Vectors. 2016;9: 1–11. doi:10.1186/s13071-016-1743-7
5. Alves F, Bilbe G, Blesson S, Goyal V, Monnerat S, Mowbray C, et al. Recent Development of Visceral Leishmaniasis Treatments: Successes, Pitfalls, and Perspectives. Clin Microbiol Rev. 2018;31: 1–30. doi:10.1128/CMR.00048-18
6. Sundar S, Singh A. Chemotherapeutics of visceral leishmaniasis: Present and future developments. Parasitology. 2018;145: 481–489. doi:10.1017/S0031182017002116
7. Croft SL, Sundar S, Fairlamb AH. Drug resistance in leishmaniasis. Clin Microbiol Rev. 2006;19: 111–126. doi:10.1128/CMR.19.1.111-126.2006
8. Sundar S, Jaya J. Liposomal amphotericin B and leishmaniasis: Dose and response. J Glob Infect Dis. 2010;2: 159. doi:10.4103/0974-777x.62886

For peer review only - http://bmjopen.bmj.com/site/about/guidelines.xhtml

9. Gonzalez-Fajardo L, Fernández OL, McMahon-Pratt D, Saravia NG. Ex Vivo Host and Parasite Response to Antileishmanial Drugs and Immunomodulators. PLoS Negl Trop Dis. 2015;9: 1–19. doi:10.1371/journal.pntd.0003820

10. Ponte-Sucre A, Gamarro F, Dujardin JC, Barrett MP, López-Vélez R, García-Hernández R, et al. Drug resistance and treatment failure in leishmaniasis: A 21st century challenge. PLoS Negl Trop Dis. 2017;11: 1–24. doi:10.1371/journal.pntd.0006052

11. Gorski S, Collin SM, Ritmeijer K, Keus K, Gatluak F, Mueller M, et al. Visceral leishmaniasis relapse in Southern Sudan (1999-2007): A retrospective study of risk factors and trends. PLoS Negl Trop Dis. 2010;4. doi:10.1371/journal.pntd.0000705

12. Cota GF, de Sousa MR, Rabello A. Predictors of visceral leishmaniasis relapse in hiv-infected patients: A systematic review. PLoS Negl Trop Dis. 2011;5: 1–8. doi:10.1371/journal.pntd.0001153

13. Burza S, Sinha PK, Mahajan R, Lima MA, Mitra G, Verma N, et al. Risk Factors for Visceral Leishmaniasis Relapse in Immunocompetent Patients following Treatment with 20 mg/kg Liposomal Amphotericin B (Ambisome) in Bihar, India. PLoS Negl Trop Dis. 2014;8: 44. doi:10.1371/journal.pntd.0002536

14. Ostyn B, Hasker E, Dorlo TPC, Rijal S, Sundar S, Dujardin JC, et al. Failure of miltefosine treatment for visceral leishmaniasis in children and men in South-East Asia. PLoS One. 2014;9. doi:10.1371/journal.pone.0100220

15. Sundar S, Singh A, Agrawal N, Chakravarty J. Effectiveness of single-dose liposomal amphotericin b in visceral leishmaniasis in Bihar. Am J Trop Med Hyg. 2019;101: 795–798. doi:10.4269/ajtmh.19-0179

16. Takele Y, Mulaw T, Adem E, Shaw CJ, Franssen SU, Womersley R, et al. Immunological factors, but not clinical features, predict visceral leishmaniasis relapse in patients co-

For peer review only - http://bmjopen.bmj.com/site/about/guidelines.xhtml

infected with HIV. Cell Reports Med. 2022;3. doi:10.1016/j.xcrm.2021.100487

17. Riley RD, Lambert PC, Abo-Zaid G. Meta-analysis of individual participant data: Rationale, conduct, and reporting. BMJ. 2010;340: c221. doi:10.1136/bmj.c221

18. Rijal S, Ostyn B, Uranw S, Rai K, Bhattarai NR, Dorlo TPC, et al. Increasing failure of miltefosine in the treatment of kala-azar in nepal and the potential role of parasite drug resistance, reinfection, or noncompliance. Clin Infect Dis. 2013;56: 1530–1538. doi:10.1093/cid/cit102

19. Sundar S, Singh A, Rai M, Chakravarty J. Single-dose indigenous liposomal amphotericin B in the treatment of Indian visceral leishmaniasis: A phase 2 study. Am J Trop Med Hyg. 2015;92: 513–517. doi:10.4269/ajtmh.14-0259

20. Singh-Phulgenda S, Rashan S, Harriss E, Dahal P, Naylor C, Brack M, et al. Infectious diseases data observatory (IDDO) visceral leishmaniasis library of clinical therapeutic studies: A protocol for a living systematic review of clinical studies. Wellcome Open Res. 2022;7: 155. doi:10.12688/wellcomeopenres.17739.1

21. Bush JT, Wasunna M, Alves F, Alvar J, Olliaro PL, Otieno M, et al. Systematic review of clinical trials assessing the therapeutic efficacy of visceral leishmaniasis treatments: A first step to assess the feasibility of establishing an individual patient data sharing platform. PLoS Negl Trop Dis. 2017;11: e0005781. doi:10.1371/journal.pntd.0005781

22. Singh-Phulgenda S, Rashan S, Harriss E, Prabin D, Naylor C, Brack M, et al. Infectious Diseases Data Observatory (IDDO) Visceral Leishmaniasis Library of Clinical Therapeutic Studies : A Protocol for a Living Systematic Review of Clinical Studies. In: Research Square PREPRINT (Version 1). 2022. doi:https://doi.org/10.21203/rs.3.rs-1159661/v1 License:

23. Infectious Diseases Data Observatory. Visceral leishmaniasis Data Inventory. In:

For peer review only - http://bmjopen.bmj.com/site/about/guidelines.xhtml

iddo.org [Internet]. 2023 [cited 27 Feb 2023]. Available: https://www.iddo.org/document/visceral-leishmaniasis-data-inventory

24. Infectious Diseases Data Observatory. New global collaboration developing standards for standardised data collection across the VL research community. 2020 [cited 28 Feb 2023]. Available: https://www.iddo.org/news/new-global-collaboration-developing-standards-standardised-data-collection-across-vl-research

25. Chulay JD, Bryceson ADM. Quantitation of amastigotes of Leishmania donovani in smears of splenic aspirates from patients with visceral leishmaniasis. Am J Trop Med Hyg. 1983;32: 475–479. doi:10.4269/ajtmh.1983.32.475

26. Srivastava P, Dayama A, Mehrotra S, Sundar S. Diagnosis of visceral leishmaniasis. Trans R Soc Trop Med Hyg. 2011;105: 1–6. doi:10.1016/j.trstmh.2010.09.006

27. Sudarshan M, Weirather JL, Wilson ME, Sundar S. Study of parasite kinetics with antileishmanial drugs using real-time quantitative PCR in Indian visceral leishmaniasis. J Antimicrob Chemother. 2011;66: 1751–1755. doi:10.1093/jac/dkr185

28. Sundar S, Singh OP. Molecular Diagnosis of Visceral Leishmaniasis. Mol Diagnosis Ther. 2018;22: 443–457. doi:10.1007/s40291-018-0343-y

29. Dorlo TPC, Rijal S, Ostyn B, Vries PJ De, Singh R, Bhattarai N, et al. Failure of Miltefosine in Visceral Leishmaniasis Is Associated With Low Drug Exposure. J Infect Dis. 2014;210: 146–153. doi:10.1093/infdis/jiu039

30. Burke DL, Ensor J, Riley RD. Meta-analysis using individual participant data: one-stage and two-stage approaches, and why they may differ. Stat Med. 2017;36: 855–875. doi:10.1002/sim.7141

31. Riley RD, Moons KGM, Debray TPA. IPD Meta-Analysis for Prognostic Factor Research. In: Richard D. Riley, Jayne F. Tierney LAS, editor. Individual Participant Data Meta-

For peer review only

For peer review only - http://bmjopen.bmj.com/site/about/guidelines.xhtml

Analysis: A Handbook for Healthcare Research. John Wiley & Sons Ltd; 2021. pp. 421–445. doi:10.1002/9781119333784.ch16

32. Heinze G, Wallisch C, Dunkler D. Variable selection – A review and recommendations for the practicing statistician. Biometrical J. 2018;60: 431–449. doi:10.1002/bimj.201700067

33. Sauerbrei W, Royston P, Binder H. Selection of important variables and determination of functional form for continuous predictors in multivariable model building. Stat Med. 2007;26: 5512–5528. doi:10.1002/sim.3148

34. White IR, Royston P, Wood AM. Multiple imputation using chained equations: Issues and guidance for practice. Stat Med. 2011;30: 377–399. doi:10.1002/sim.4067

35. World Health Organization. WHO guidelines for the treatment of visceral leishmaniasis in HIV co-infected patients in East Africa and South-East Asia. World Health OrganizationWorld Health Organization. Geneva, Switzerland; 2022.

36. Riley RD, Debray TPA, Fisher D, Hattle M, Marlin N, Hoogland J, et al. Individual participant data meta-analysis to examine interactions between treatment effect and participant-level covariates: Statistical recommendations for conduct and planning. Stat Med. 2020;39: 2115–2137. doi:10.1002/sim.8516

37. R Core Team. R: A language and environment for statistical computing. Vienna, Austria; 2021. Available: https://www.r-project.org/.

38. StataCorp. Stata Statistical Software: Release 17. College Station, TX; 2021.

39. Sterne JAC, Savović J, Page MJ, Elbers RG, Blencowe NS, Boutron I, et al. RoB 2: A revised tool for assessing risk of bias in randomised trials. BMJ. 2019;366: 1–8. doi:10.1136/bmj.l4898

40. Sterne JA, Hernán MA, Reeves BC, Savović J, Berkman ND, Viswanathan M, et al.

For peer review only

For peer review only - http://bmjopen.bmj.com/site/about/guidelines.xhtml

ROBINS-I: A tool for assessing risk of bias in non-randomised studies of interventions. BMJ. 2016;355: 4–10. doi:10.1136/bmj.i4919

41. Stewart LA, Clarke M, Rovers M, Riley RD, Simmonds M, Stewart G, et al. Preferred reporting items for a systematic review and meta-analysis of individual participant data: The PRISMA-IPD statement. JAMA - J Am Med Assoc. 2015;313: 1657–1665. doi:10.1001/jama.2015.3656

42. Infectious Diseases Data Observatory. Visceral leishmaniasis research agenda. In: 2019 [Internet]. [cited 28 Feb 2023]. Available: https://www.iddo.org/vl/research/research-agenda

43. Rijal S, Sundar S, Mondal D, Das P, Alvar J, Boelaert M. Eliminating visceral leishmaniasis in South Asia: The road ahead. BMJ. 2019;364: k5224.

44. World Health Organization. Regional Strategic Framework for Elimination of Kala-azar from the South-East Asia Region: 2022-2026. New Delhi; 2022.

45. Naylor-leyland G, Collin SM, Gatluak F, Id MDB, Alves F, Mullahzada AW, et al. The increasing incidence of visceral leishmaniasis relapse in South Sudan: A retrospective analysis of field patient data from 2001 – 2018. PLoS Negl Trop Dis. 2022; 1–14. doi:10.1371/journal.pntd.0010696

46. Singh-Phulgenda S, Dahal P, Ngu R, Maguire BJ, Hawryszkiewycz A, Rashan S, et al. Serious adverse events following treatment of visceral leishmaniasis: A systematic review and meta-analysis. PLoS Negl Trop Dis. 2021;15: e0009302. doi:10.1371/journal.pntd.0009302

For peer review only

For peer review only - http://bmjopen.bmj.com/site/about/guidelines.xhtml

BMJ Open

Host, parasite, and drug determinants of clinical outcomes following treatment of visceral leishmaniasis: a protocol for individual participant data meta-analysis

Journal:	*BMJ Open*
Manuscript ID	bmjopen-2023-074679.R1
Article Type:	Protocol
Date Submitted by the Author:	23-Aug-2023
Complete List of Authors:	Kumar, Rishikesh; Rajendra Memorial Research Institute of Medical Sciences (RMRIMS)<br>Dahal, Prabin; Infectious Diseases Data Observatory (IDDO); University of Oxford, Centre for Tropical Medicine and Global Health, Nuffield Department of Clinical Medicine<br>Singh-Phulgenda, Sauman; Infectious Diseases Data Observatory (IDDO); University of Oxford, Centre for Tropical Medicine and Global Health, Nuffield Department of Clinical Medicine<br>Siddiqui, Niyamat; Rajendra Memorial Research Institute of Medical Sciences (RMRIMS)<br>Munir, Abdalla; Infectious Diseases Data Observatory; University of Oxford Centre for Tropical Medicine and Global Health, Centre for Tropical Medicine and Global Health, Nuffield Department of Clinical Medicine<br>Naylor, Caitlin; Infectious Diseases Data Observatory (IDDO); University of Oxford, Centre for Tropical Medicine and Global Health, Nuffield Department of Clinical Medicine<br>Wilson, James; Infectious Diseases Data Observatory (IDDO); University of Oxford, Centre for Tropical Medicine and Global Health, Nuffield Department of Clinical Medicine<br>Buck, Gemma; Infectious Diseases Data Observatory (IDDO); University of Oxford, Centre for Tropical Medicine and Global Health, Nuffield Department of Clinical Medicine<br>Rahi, Manju; ICMR, Epidemiology and Communicable Diseases<br>Malaviya, Paritosh; Banaras Hindu University, Department of Medicine, Institute of Medical Sciences<br>Alves, Fabiana; Drugs for Neglected Disease Initiative<br>Sundar, Shyam; Banaras Hindu Univ<br>Ritmeijer, Koert; Medecins Sans Frontieres, Public Health Department<br>Stepniewska, Kasia; Infectious Diseases Data Observatory (IDDO); University of Oxford, Centre for Tropical Medicine and Global Health, Nuffield Department of Clinical Medicine<br>Guérin, Philippe J.; Infectious Diseases Data Observatory/University of Oxford<br>Pandey, Krishna; Rajendra Memorial Research Institute of Medical Sciences (RMRIMS)
**Primary Subject Heading**:	Global health

Secondary Subject Heading:	Epidemiology, Global health, Infectious diseases, Public health
Keywords:	EPIDEMIOLOGY, Neglected Diseases, INFECTIOUS DISEASES, PUBLIC HEALTH, Tropical medicine < INFECTIOUS DISEASES, Epidemiology < INFECTIOUS DISEASES

SCHOLARONE™
Manuscripts

Title: Host, parasite, and drug determinants of clinical outcomes following treatment of visceral leishmaniasis: a protocol for individual participant data meta-analysis

Rishikesh Kumar [1], Prabin Dahal [2,3§], Sauman Singh-Phulgenda [2,3], Niyamat Ali Siddiqui [1], Abdalla Munir [2,3], Caitlin Naylor [2,3], James Wilson[2,3], Gemma Buck [2,3], Manju Rahi [4], Paritosh Malaviya [5], Fabiana Alves [6], Shyam Sundar [5], Koert Ritmeijer[7], Kasia Stepniewska [2,3], Philippe J Guerin[2,3], Krishna Pandey [1§]

[1] ICMR – Rajendra Memorial Research Institute of Medical Sciences (RMRIMS), Patna, India

[2]Infectious Diseases Data Observatory (IDDO), Oxford, UK

[3]Centre for Tropical Medicine and Global Health, Nuffield Department of Clinical Medicine, University of Oxford, Oxford, UK

[4]Indian Council of Medical Research (ICMR), New Delhi, India

[5]Infectious Disease Research Laboratory, Department of Medicine, Institute of Medical Sciences, Banaras Hindu University, Varanasi, India

[6]Drugs for Neglected Diseases initiative, Geneva, Switzerland

[7]Médecins Sans Frontières, Amsterdam, Netherlands

[§]*Correspondence: drkrishnapandey@gmail.com, prabin.dahal@ndm.ox.ac.uk*

For peer review only

For peer review only - http://bmjopen.bmj.com/site/about/guidelines.xhtml

Abstract

Introduction: Visceral leishmaniasis (VL) is a parasitic disease with an estimated 30,000 new cases occurring annually. There is an observed variation in the efficacy of the current first line therapies across different regions. Such heterogeneity could be a function of host, parasite, and drug factors. An individual participant data meta-analysis (IPD-MA) is planned to explore the determinants of treatment outcomes.

Methods and analysis: The Infectious Diseases Data Observatory (IDDO) VL living systematic review (IDDO VL LSR) library is an open-access resource of all published therapeutic studies in VL since 1980 (PROSPERO registration: CRD42021284622). For this current review, the search includes all clinical trials published between 1st of Jan 1980 and 2nd of May 2021. Studies indexed in the IDDO VL LSR library were screened for eligibility for inclusion in this IPD-MA. Corresponding authors and principal investigators of the studies meeting the eligibility criteria for inclusion were invited to be part of the collaborative IPD-MA. Authors agreeing to participate in this collaborative research were requested to share the IPD using the IDDO (IDDO) VL data platform. The IDDO VL data platform currently holds datasets from clinical trials standardised to a common data format and provides a unique opportunity to identify host, parasite, and drug determinants of treatment outcomes. Multivariable regression models will be constructed to identify determinants of therapeutic outcomes using generalised linear mixed effects models accounting for within-study site clustering.

Ethics and dissemination: This IPD-MA meets the criteria for waiver of ethical review as defined by the Oxford Tropical Research Ethics Committee (OxTREC) granted to IDDO, as the research consists of secondary analysis of existing anonymised data (Exempt granted on 29th

For peer review only

For peer review only - http://bmjopen.bmj.com/site/about/guidelines.xhtml

March 2023, OxTREC REF: Infectious Diseases Data Observatory (IDDO)). The results of this IPD-MA will be disseminated at conferences, IDDO website and any peer-reviewed publications. All publications will be open source. Findings of this research will be critically important for the control programmes at regional/global levels, policy makers and groups developing new VL treatments.

PROSPERO registration: The systematic review component of the IPD-MA has the following PROSPERO registration: CRD42021284622.

Author Email:

RK drkumarrishikesh@gmail.com
PD prabin.dahal@iddo.org
SSP sauman.singh@iddo.org
NAS niyamatalisiddiqui@yahoo.com
AM abdalla.munir@iddo.org
CN caitlin.naylor@iddo.org
JW james.wilson@iddo.org
GB gemma.buck@iddo.org
MR drmanjurahi@gmail.com
PM paritosh_malaviya@yahoo.com
FA falves@dndi.org
SS drshyamsundar@hotmail.com
KR koert.ritmeijer@amsterdam.msf.org
KS kasia.stepniewska@iddo.org
PJG philippe.guerin@iddo.org
KP drkrishnapandey@yahoo.com

Keywords

For peer review only

For peer review only - http://bmjopen.bmj.com/site/about/guidelines.xhtml

Visceral Leishmaniasis; Therapeutic Outcomes; Statistical Analysis Plan, meta-analysis, individual participant data; IPD meta-analysis; relapse

Word count: 3478 (main text); abstract (255)

Strengths and limitations of this study

- In any single Visceral Leishmaniasis clinical trial, only few relapses are observed which limits the ability to identify predictors associated with it. An individual participant data meta-analysis (IPD-MA) will increase the statistical power to detect the predictors and moderators of treatment outcome.

- The identification of studies eligible for inclusion in the IPD-MA has been made through a comprehensive literature search of all published studies since 1980 with pre-defined inclusion-exclusion criteria. However, retrieval of data from trials published prior to 2000 can be a major challenge.

- This IPD-MA will utilise the VL repository of individual participant data hosted by Infectious Diseases Data Observatory (The IDDO VL data platform). A major strength of this study is that data on the IDDO VL data platform is harmonised to a common standard based on an extensive consultation with the VL research community.

- A particular scientific challenge is that distinction of relapse from re-infection is seldom carried out in VL therapeutic trials and hence won't be considered in this analysis.

For peer review only - http://bmjopen.bmj.com/site/about/guidelines.xhtml

For peer review only

For peer review only

For peer review only - http://bmjopen.bmj.com/site/about/guidelines.xhtml

Introduction

Visceral leishmaniasis (VL), also known as kala-azar, is a parasitic disease with anthroponotic and zoonotic modes of transmission [1]. The disease is characterised by prolonged fever, cachexia, splenomegaly, hepatomegaly and anaemia. The annual global incidence of VL is estimated at 30,000 cases, with only 25-45% of the cases reported to the World Health Organization [2]. The disease has an outbreak potential and linked with war, conflicts and climate change, and if left untreated, the disease is fatal in over 95% of the cases [2–4].

The efficacy of antileishmanial regimens in VL varies across geographical regions. For example, single dose liposomal amphotericin B (L-AmB) has a demonstrated high efficacy (≥94%) in the Indian sub-continent while its efficacy is sub-optimal (58%) in East Africa [5,6]. There are high levels of resistance against antimony-based drugs in India, although it continues to be used in a combination regimen with paromomycin as a first line therapy in East Africa [5,6]. The underlying reasons for such variation are not fully understood but the observed therapeutic response is likely multifactorial and may be a function of drug resistance, variation in the pharmacokinetic and pharmacodynamic properties of the drugs used and the underlying host immunity [7–10].

Several studies have reported on the determinants of therapeutic outcomes in studies conducted across different geographical regions and patient populations [11–16]. Additionally, the heterogeneity introduced by variation in study design and conduct leads to challenges in reliably comparing the effect measures across the studies. A major challenge in reliably assessing risk factors of therapeutic outcomes is that at individual study level, the number of relapses is often small [17], which limits the precision of the estimated effect.

For peer review only - http://bmjopen.bmj.com/site/about/guidelines.xhtml

Individual participant data (IPD) meta-analysis (IPD-MA) of existing studies can overcome this limitation by increasing the effective sample size and allows for greater power in detecting differential treatment effects across individuals in trials [18].

Objectives

The objectives of this IPD-MA are:

- To identify host, parasite, and drug determinants of initial cure after the completion of treatment schedule
- To identify host, parasite, and drug determinants of definitive cure at 6 months after treatment completion
- To identify host, parasite, and drug determinants of relapse at 6 months after treatment completion
- To identify host, parasite, and drug determinants of mortality outcome at any time point during the study

Primary and secondary endpoints relating to these objectives are defined in the outcomes section.

For peer review only - http://bmjopen.bmj.com/site/about/guidelines.xhtml

Methods and analysis: Patients, interventions and outcomes

PICOT statement

Population: Any patient enrolled in an interventional study with a confirmed or suspected diagnosis of VL defined by serological and/or parasitological testing.

Interventions: Any antileishmanial therapy

Comparator: Not restricted by the use of a comparator drug

Outcome: At least one of the following outcomes reported: initial cure, definitive cure, relapse, or mortality

Time: Studies published on or after 1980

Criteria for study eligibility

The following eligibility criteria must be fulfilled for study inclusion:

- Prospective clinical efficacy studies on patients with confirmed or suspected VL either using microscopy/serology/molecular method (i.e. clinical diagnosis followed by a confirmatory method)
- Information on constituent drug(s), dose and duration of treatment regimen is available
- At least one of the following clinical outcomes are measured: initial cure, definitive cure, relapse or mortality

For peer review only - http://bmjopen.bmj.com/site/about/guidelines.xhtml

Criteria for patient eligibility

The following minimum information are required for inclusion of a patient in the IPD meta-analysis:

- Details of antileishmanial treatment(s) administered
- Baseline information on age and gender
- Outcome is recorded

Outcomes

Outcomes and definitions:

Primary endpoint

The primary endpoint adopted is cure at 6-months following treatment completion. This is commonly referred to as 'definitive' or 'final' cure. The precise definition and timing of definitive cure will vary according to study design. As a minimum, it is expected that a definitive cure is achieved if the patient remains alive and is no longer be manifesting symptoms and/or signs of visceral leishmaniasis.

Further analysis of this endpoint will be carried out using a stricter definition of definitive cure, defined as: achievement of initial cure (as defined below) and no subsequent rescue treatment or evidence of clinical or parasitological relapse, and no death associated with VL during the 6-months following treatment completion.

Secondary endpoints:

The following secondary endpoints are adopted:

For peer review only

For peer review only - http://bmjopen.bmj.com/site/about/guidelines.xhtml

Initial cure

Initial cure is defined at the time of initial assessment adopted in the original study after completion of the therapy along with clinical improvement. The timing of initial cure assessment would typically take place within 28 days of treatment completion but this varies slightly across studies.

Further analysis of this endpoint will be carried out using a stricter definition of initial cure, defined as: cure at day 28 or at time-point defined in the original study following treatment, defined as clinical improvement of VL, absence of parasites in the spleen or bone marrow using microscopy) and no rescue therapy on or before day 28 or before the timepoint of initial cure assessment adopted in the original study.

Relapse

Relapse is defined as the recurrence of signs and symptoms of VL at any time point during the study follow-up among those who had achieved initial cure. The assessment typically takes place at 6 months but this varies slightly across studies.

Tissue aspirates for confirming parasitological presence are usually carried out based on clinical suspicion of relapse during the follow-up [19,20]. Since tissue aspiration for defining relapse won't be carried out in all the studies, further analysis of this endpoint will be undertaken by considering the methodology used to define relapse.

Mortality

This is defined as deaths reported as related to the study interventions or due to the progressive worsening of the disease itself at any time during the study. Further analysis of this endpoint will be carried out by considering all-cause mortality.

For peer review only

For peer review only - http://bmjopen.bmj.com/site/about/guidelines.xhtml

197 ***Variation is time-points***

198 The assessment of initial cure and definitive cure is usually undertaken at 28-days and 180-days post treatment. However, the exact time of these assessment will vary across the studies. Assessments undertaken between 15 to 60 days will be considered as time of initial cure. For assessments of definitive cure and relapse, assessments made between 150 to 210 days will be considered as 180 days.

203

204 ## Statistical methods

205 **Identification of relevant studies**

206 Eligible studies were identified from the infectious diseases data observatory (IDDO) library of all prospective therapeutic studies that has systematically indexes all published therapeutic efficacy studies in VL published from 1980 onwards [21]. The IDDO VL clinical trials library is based on a living systematic review and the database is continually updated every six months in accordance with the Preferred Reporting Items for Systematic-Reviews and Meta-Analyses (PRISMA) guidelines. The trial library indexes publications identified from the following databases: PubMed, Embase, Scopus, Web of Science, Cochrane, clinicaltrials.gov, WHO ICTRP, Global Index Medicus, IMEMR, IMSEAR, and LILACS. For this current review, the search includes all clinical trials published between 1st of Jan 1980 and 2nd of May 2021. Details of the search strategy adopted is described elsewhere [21]. The search details are presented in a online supplemental file S1. Studies indexed in the IDDO VL library were eligible for inclusion in this review if they meet the inclusion and exclusion criteria outlined above. This review is not limited by language.

For peer review only - http://bmjopen.bmj.com/site/about/guidelines.xhtml

For peer review only

Collation of IPD

Authors (principal investigators, corresponding authors) of the studies in the IDDO VL LSR library eligible for the inclusion in this IPD-MA were invited to be part of this collaborative research. Researchers agreeing to the terms and conditions of the submission were then requested to upload IPD to the IDDO VL data platform through a secured web portal [22]. Data in the IDDO VL platform are fully pseudonymised to protect personal information and patient privacy.

Data management

Raw data from individual studies shared with IDDO VL data platform are currently being standardised using the Clinical Data Interchange Standards Consortium (CDISC) compliant curation standards [23]. If required, investigators will be further contacted to clarify questions that arise during data curation and analysis, and individual study protocols will be requested. On standardisation, the data are stored in a relational database containing information on drug regimen, parasitological, clinical, and haematological assessments, and therapeutic outcomes.

For peer review only

For peer review only - http://bmjopen.bmj.com/site/about/guidelines.xhtml

Statistical methods for primary and secondary outcomes

Variables considered for regression modelling: The following variables will be considered for inclusion in the analysis of primary and secondary endpoints:

Host variables

The following host variables are considered: age, gender, body weight, nutritional status, comorbidity status (such as HIV), and duration of illness prior to study enrolment. The following baseline clinical measurements will be considered: haemoglobin/anaemia status, spleen size, immunological biomarkers (CD4+ count), alanine transaminase (ALT) and aspartate transaminase (AST), platelets and neutrophils. If data are available, the following baseline characteristics will be considered: history of blood transfusion, bilirubin, creatinine, urea, and albumin concentrations.

Parasite variables

The following parasite related baseline factors will be considered: parasite load and information regarding the nature of infection (primary vs. previously treated cases). Any cases described as previously untreated (or "fresh") cases for leishmaniasis will be considered as primary VL. The enumeration of parasite density is usually carried out by evaluating bone marrow or splenic aspirates or through quantitation of parasites in unit quantity of specimen or parasite DNA equivalence per PCR reaction across studies [24–27]; the Chulay-Bryceson scale being the most commonly adopted [24]. The methodology used for parasite gradation will be considered in the analysis.

Drug variables

For peer review only - http://bmjopen.bmj.com/site/about/guidelines.xhtml
For peer review only

257 The following drug related variables will be considered for inclusion in analysis: 258 mg/kg total dose (or target dose) administered [28], treatment duration, whether the 259 regimen was monotherapy or a combination therapy, mode of drug administration 260 (intravenous, intramuscular or oral), administration of regimen either as a single dose or as 261 a multiple doses, direct or indirect observations of drug administration for oral medications. 262 The dosage of antileishmanial interventions will be determined and expressed as dose per 263 unit body weight (e.g., total mg/kg dosage administered) and will be considered separately 264 for each drug regimens.

265 **Study level variables**

266 The following study or arm level variables will be considered in the analysis of 267 primary and secondary endpoints: country, study site, and calendar year of the study 268 conduct.

269 **Geographical variation in treatment response**

270 There is a known regional variation in treatment response in VL [5], along with 271 differences in patient characteristics and treatment guidelines. Therefore, a separate 272 analysis will be undertaken within each geographical region to construct the univariable and 273 multivariable regression models for the primary and secondary outcomes.

274 **Statistical analysis and reporting**

275 **Descriptive summary**

276 A summary of included studies will be presented with respect to study design, study 277 location, year of study conduct, characteristics of study population, duration of follow-up,

For peer review only

For peer review only - http://bmjopen.bmj.com/site/about/guidelines.xhtml

details of drug regimens including supervision of drug administration, and methodological details adopted for disease confirmation and treatment outcomes.

Summary of baseline characteristics of the patients included in the analysis will be presented by geographical region and overall. These include: age, weight, parasite grade at enrolment, temperature, haemoglobin (or haematocrit) concentration, anaemia and severe anaemia status, spleen size, description of severity of infection (severe/mild/uncomplicated) if available, total mg/kg dose for each treatment, and supervision of drug administration. The distribution of the baseline characteristics will be summarised either as a proportion for categorical variables, as mean (with standard deviation) or median (with interquartile range) for continuous variables.

Analysis of the primary endpoint:

A mixed effects logistic regression model will be used for identifying the risk factors for definitive cure in a one-stage IPD-MA. Given a relatively low number of expected events (for relapse) in each included study [17], to avoid imprecise estimates from small events and continuity corrections, a one-stage IPD approach is preferred [29]. A random intercept regression model will be considered with study sites specified as random effects to account for within-site clustering of the patients (as some of the trials are multicentre studies).

If information regarding the time of occurrence of event is available, Kaplan-Meier (K-M) method will be used to estimate the probability of definitive cure at the end of the study follow-up, and a Cox regression model will be considered with study sites fitted as a frailty term to account for between-study site heterogeneity. The following outcomes will

For peer review only - http://bmjopen.bmj.com/site/about/guidelines.xhtml

be censored in the survival analysis: lost to follow-up, death due to causes adjudicated as unrelated to the study drug, voluntary withdrawal from the study.

Multivariable modelling

Core predictor set

The following set of variables are the risk factors reported in clinical literature for treatment outcomes in VL: age, sex, baseline parasite density, and HIV co-infection. These variables along with the drug regimen will form the minimal adjustment set for assessment of other risk factors and will be kept in the regression model regardless of statistical significance [30].

Assessment of other predictors and considerations for multivariable modelling

The association between the remaining candidate predictors and therapeutic outcomes will be assessed by adjusting for the core predictor sets identified. Multivariable model construction will follow the recommendations of Heinze et al (2017) [31]. Nested models will be compared by assessing the change in log-likelihood estimates and Akaike's information criterion (AIC) will be used for comparing competing non-nested models. The functional form of the continuous variables will be determined using multivariable fractional polynomials [32] or restricted cubic splines [30]. Stability investigations will be undertaken to account for uncertainty introduced in multivariable modelling through bootstrap resampling [31].

Missing data

The proportion of missing observation for each of the variables considered in the analysis will be summarised and any missingness patterns will be explored. Multiple

For peer review only - http://bmjopen.bmj.com/site/about/guidelines.xhtml

imputation [33], which assumes missing at random mechanism for missingness, will be undertaken to handle missing observations. Construction of the imputation model will include all the variables in the target analysis (i.e., all included exposures, outcome in the target analysis), and additional auxiliary variables including any interaction terms and nonlinear associations. The number of imputations will be determined based on the fraction of missing information. The target analysis will then be carried out in each of the completed (observed plus imputed values) datasets and the estimates will be combined across the imputed datasets using Rubin's combination rules [33].

Subgroup analysis

Patients living with HIV who are treated for VL typically have worse outcomes and higher mortality risk than those who are not living with HIV [34,35]. Although, usually an exclusion criterion of therapeutic trials, a separate sub-group analysis will be carried out among patients with defined VL-HIV coinfections (data permitting). An interaction between treatment and HIV status (treatment-covariate interaction) will therefore be considered; within-study interaction will be separated from the between-study interaction by centering the covariates [36].

Sensitivity analyses

Stability investigations will be undertaken to account for uncertainty introduced in multivariable modelling through bootstrap resampling. The robustness of the derived estimates and their variance will be summarised using the recommendations in Heinze et al (2017) [31]. The influence of each of the studies towards the estimated regression coefficients will be assessed by removing each study at a time and estimating the coefficient of variation for the parameter estimates.

For peer review only - http://bmjopen.bmj.com/site/about/guidelines.xhtml

Analysis of secondary endpoints

Initial cure

The proportion of patients achieving initial cure (as defined previously) in each of the studies will be presented. The construction of univariable and multivariable regression models will follow the same approach as for the primary endpoint.

Relapse

The proportion of patients achieving relapse (as defined previously) in each of the studies will be presented. The construction of univariable and multivariable regression models will follow the same approach as for the primary endpoint.

Mortality

The proportion of deaths reported in each of the studies will be presented. The construction of univariable and multivariable regression models will follow the same approach as for the primary endpoint.

Software

All analyses will be carried out using R software or Stata 17 software [37,38]. Use of any other data analysis tools will not change the statistical analysis plan.

Risk of bias assessment in included studies and in the IPD-MA

To examine the risk of bias in included studies, the Cochrane risk of bias tool (RoB 2) for randomised studies [39]. Risk of bias in non-randomised studies will be carried out by assessing the pre-intervention, at intervention and post-intervention domains as outlined in using ROBINS-I tool [40].

For peer review only - http://bmjopen.bmj.com/site/about/guidelines.xhtml

For peer review only

To examine the risk of bias in IPD-MA, the first four domains of the quality In prognosis studies (QUIPS) tool and the first three domains of the prediction model risk of bias assessment tool (PROBAST) will be considered as recommended in Riley et al (2021) [30]. The relevant domains from the QUIPS checklist are study participation, study attrition, prognostic factor measurement, and outcome measurement, and the relevant domains from PROBAST checklist are patient selection, prognostic factors, and outcomes. Two reviewers will independently assess the risk of bias in the studies included in the analysis.

Assessment of risk of potential bias in missing studies

Despite best possible efforts, it is anticipated that raw data from all the identified studies will not be available. The characteristics of patient population and study meta-data from the missing studies will be summarised to explore if the missing studies are systematically different from the studies that are included in the meta-analysis. A two-stage IPD may be conducted if sufficient details (or any covariate adjustment) are reported in the original studies.

For peer review only

For peer review only - http://bmjopen.bmj.com/site/about/guidelines.xhtml

Dissemination plans

Ethics and dissemination

This IPD-MA meets the criteria for waiver of ethical review as defined by the Oxford Tropical Research Ethics Committee (OxTREC) granted to IDDO, as the research consists of secondary analysis of existing anonymised data (Exempt granted on 29th March 2023, OxTREC REF: Infectious Diseases Data Observatory (IDDO)). Ethical approval was granted to each study included in this pooled analysis by their respective ethics committees. This IPD-MA will address research questions similar to that of included studies. Findings of this IPD-MA will be reported in open-access, peer-reviewed journals following the PRISMA-IPD guidelines [41].

Patient and public involvement

The research questions considered in this IPD-MA is based on a research agenda developed by the global VL research community [42]. The design and development of this IPD-MA were done by the study authors only and no patient was involved at any stage.

Further development of statistical analysis plan

Major statistical analyses have been included in this plan. Amendments to the current plan or additional statistical analyses may be required as data accrual is in progress and will be transparently reported in subsequent reports of the results.

For peer review only - http://bmjopen.bmj.com/site/about/guidelines.xhtml

Discussion

Global commitment over the past few decades has led to significant progress in the control and elimination of VL. In the Indian subcontinent (ISC) the VL burden has greatly reduced in the past 15 years [43,44]. Availability of effective antileishmanial treatment is the cornerstone for achieving and sustaining elimination in the ISC. As the overall incidence of VL is falling, a growing proportion of the total reported cases in the region is attributed to relapses [44]. A recent report from South Sudan has also indicated increasing incidence of relapses over the past two decades [45]. This is an important public health concern as patients with relapse are predisposed to further relapse, especially among those with HIV co-infections [12]. Increasingly large proportion of VL patients have been found to present with HIV co-infections in Brazil (0.7% in 2001 to 8.5% in 2012), India (0.88% in 2000 to 4.19% in 2020) and Northern Ethiopia (15-35%)[35]. A study in India found that over half of those with HIV co-infections were unaware of their status [46]. This presents an important challenge to the ongoing control and elimination efforts as patients with VL-HIV co-infections typically have worse outcomes and higher mortality risk than those who are not living with HIV [34,35]. VL-HIV patients are also recognised as an important reservoir of transmission as the co-infected patients are predisposed to multiple relapses, and have a high potential for infectiousness to sand-flies due to the generally high parasite loads and poses threat to the control and elimination efforts [47,48]. Therefore, the identification of host, parasite or drug related characteristics remain crucial not only for effective case management but also for disease control and elimination.

Several clinical trials and meta-analyses have highlighted the therapeutic efficacy and safety of one or more antileishmanial therapies [5,11–13,16,34]. However, risk factors

For peer review only - http://bmjopen.bmj.com/site/about/guidelines.xhtml

determining the undesirable therapeutic outcomes remain poorly understood. Underlying heterogeneities in the published studies in terms of study population, treatment regimen, outcome definitions, and study designs render difficulties in drawing a comprehensive conclusion regarding drug efficacy. In addition, the national treatment guidelines differ in terms of practices and approaches for case management. Some of these limitations could be addressed through a well-designed prospective study, which will incur a substantial logistical and financial costs to reach a critical mass required for robust investigation of the predictors of treatment relapse and remains unfeasible to achieve within short period of time. Utilising the existing datasets and undertaking a carefully planned IPD-MA can ameliorate some of these limitations [18]. We hope that this IPD-MA will provide evidence for the efficacy of different treatments in VL that can be further considered by policy makers at regional and global levels.

The identification of studies eligible for inclusion in the IPD-MA has been made through a comprehensive literature search of all published studies since 1980 with pre-defined inclusion-exclusion criteria. A major strength of this study is that data from several studies will be harmonised to a common standard based on an extensive consultation with the VL research community [23], thus allowing us to address some of the methodological sources of heterogeneity.

A major challenge is that a substantial proportion of the studies in the IDDO library were conducted prior to the year 2000; the retrieval of data from historical trials is a major challenge. A particular scientific challenge is that misclassification of re-infection as “true” relapse can potentially introduce bias in relapse estimates. A distinction of relapse and re-infection is seldom carried out in VL therapeutic trials and hence won’t be considered in this

For peer review only - http://bmjopen.bmj.com/site/about/guidelines.xhtml

IPD-MA. Evidence form a study in Nepal suggested late relapses were less likely to be new infections [19], but generalisability to other settings remains unclear. Finally, the determinants of therapeutic outcomes in this IPD-MA would be limited to the commonly assessed and available clinical-laboratory parameters across VL drug trials. Similarly, the exploration of parasitic factors is limited to parasite gradation and the nature of the infection (primary vs previously treated cases). Other important parasite factors such as *in vitro* status of drug susceptibility, their virulence, and the underlying genomic plasticity allowing parasites to undergo mutation under drug pressure[49,50] are not routinely collected in clinical trials and hence remains beyond the scope of this IPD-MA.

This IPD-MA will combine data from the studies in the IDDO VL data platform to identify the determinants of therapeutic outcomes. Findings of this research will generate important information for the control programmes at regional and global levels, policy makers and groups developing new VL treatments.

For peer review only - http://bmjopen.bmj.com/site/about/guidelines.xhtml

457 **Authors' contributions**

458 Study Conception: RK, PD, SSP, NAS, AM, CN, JW, GB, MR, PM, FA, SS, KR, KS, PJG and KP.

459 Project supervision: SSP, MR, KP, PJG.

460 Methodology: PD, NAS, SSP, SS, KR, JW, FA, PJG, KS

461 Data curation: AM, SSP, PM, JW, PD, GB

462 Project administration: SSP, CN

463 Funding acquisition: PJG

464 Resources: SSP, FA, PJG

465 Writing-original draft: RK, PD, KP, PJG, KS

466 Writing- review and editing: All authors were involved in reading and critical revision of the
467 initial draft and approved the final manuscript.

468

469 **Funding**

470 This work is funded by a Bill & Melinda Gates Foundation grant to the Infectious Diseases
471 Data Observatory, Oxford University, UK (Recipient: Prof. Philippe Guerin; ref: INV-004713).
472 Funding agency had no role in developing the protocol.

473 **Conflict of Interest**

474 None

475 **Data Availability**

476 N/A as this is an analysis protocol.

For peer review only - http://bmjopen.bmj.com/site/about/guidelines.xhtml

For peer review only

References

1. Boelaert M, Sundar S. Leishmaniasis. In: Farrar J, Hotez PJ, Junghanss T, Kang G, Lalloo D, White NJ, editors. Manson's Tropical Infectious Diseases, Twentry-third edition. Elsevier Health Sciences; 2014. pp. 631-651.e4. doi:https://doi.org/10.1016/B978-0-7020-5101-2.00048-0
2. World Health Organization. Leishmaniasis. In: WHO [Internet]. 2023 [cited 23 Aug 2023]. Available: https://www.who.int/health-topics/leishmaniasis#tab=tab_1
3. Collin S, Davidson R, Ritmeijer K, Keus K, Melaku Y, Kipngetich S, et al. Conflict and kala-azar: Determinants of adverse outcomes of kala-azar among patients in southern Sudan. Clin Infect Dis. 2004;38: 612–619. doi:10.1086/381203
4. Al-Salem W, Herricks JR, Hotez PJ. A review of visceral leishmaniasis during the conflict in South Sudan and the consequences for East African countries. Parasites and Vectors. 2016;9: 1–11. doi:10.1186/s13071-016-1743-7
5. Alves F, Bilbe G, Blesson S, Goyal V, Monnerat S, Mowbray C, et al. Recent Development of Visceral Leishmaniasis Treatments: Successes, Pitfalls, and Perspectives. Clin Microbiol Rev. 2018;31: 1–30. doi:10.1128/CMR.00048-18
6. Sundar S, Singh A. Chemotherapeutics of visceral leishmaniasis: Present and future developments. Parasitology. 2018;145: 481–489. doi:10.1017/S0031182017002116
7. Croft SL, Sundar S, Fairlamb AH. Drug resistance in leishmaniasis. Clin Microbiol Rev. 2006;19: 111–126. doi:10.1128/CMR.19.1.111-126.2006
8. Sundar S, Jaya J. Liposomal amphotericin B and leishmaniasis: Dose and response. J Glob Infect Dis. 2010;2: 159. doi:10.4103/0974-777x.62886
9. Gonzalez-Fajardo L, Fernández OL, McMahon-Pratt D, Saravia NG. Ex Vivo Host and

For peer review only

For peer review only - http://bmjopen.bmj.com/site/about/guidelines.xhtml

Parasite Response to Antileishmanial Drugs and Immunomodulators. PLoS Negl Trop Dis. 2015;9: 1–19. doi:10.1371/journal.pntd.0003820

10. Ponte-Sucre A, Gamarro F, Dujardin JC, Barrett MP, López-Vélez R, García-Hernández R, et al. Drug resistance and treatment failure in leishmaniasis: A 21st century challenge. PLoS Negl Trop Dis. 2017;11: 1–24. doi:10.1371/journal.pntd.0006052

11. Gorski S, Collin SM, Ritmeijer K, Keus K, Gatluak F, Mueller M, et al. Visceral leishmaniasis relapse in Southern Sudan (1999-2007): A retrospective study of risk factors and trends. PLoS Negl Trop Dis. 2010;4. doi:10.1371/journal.pntd.0000705

12. Cota GF, de Sousa MR, Rabello A. Predictors of visceral leishmaniasis relapse in hiv-infected patients: A systematic review. PLoS Negl Trop Dis. 2011;5: 1–8. doi:10.1371/journal.pntd.0001153

13. Burza S, Sinha PK, Mahajan R, Lima MA, Mitra G, Verma N, et al. Risk Factors for Visceral Leishmaniasis Relapse in Immunocompetent Patients following Treatment with 20 mg/kg Liposomal Amphotericin B (Ambisome) in Bihar, India. PLoS Negl Trop Dis. 2014;8: 44. doi:10.1371/journal.pntd.0002536

14. Ostyn B, Hasker E, Dorlo TPC, Rijal S, Sundar S, Dujardin JC, et al. Failure of miltefosine treatment for visceral leishmaniasis in children and men in South-East Asia. PLoS One. 2014;9. doi:10.1371/journal.pone.0100220

15. Sundar S, Singh A, Agrawal N, Chakravarty J. Effectiveness of single-dose liposomal amphotericin b in visceral leishmaniasis in Bihar. Am J Trop Med Hyg. 2019;101: 795–798. doi:10.4269/ajtmh.19-0179

16. Takele Y, Mulaw T, Adem E, Shaw CJ, Franssen SU, Womersley R, et al. Immunological factors, but not clinical features, predict visceral leishmaniasis relapse in patients co-infected with HIV. Cell Reports Med. 2022;3. doi:10.1016/j.xcrm.2021.100487

For peer review only

For peer review only - http://bmjopen.bmj.com/site/about/guidelines.xhtml

17. Chhajed R, Dahal P, Singh-Phulgenda S, Brack M, Naylor C, Sundar S, et al. Estimating the proportion of relapse following treatment of Visceral Leishmaniasis: Meta-analysis using Infectious Diseases Data Observatory (IDDO) living systematic review. In: Available at SSRN: https://ssrn.com/abstract=4464863. 2023. doi:http://dx.doi.org/10.2139/ssrn.4464863

18. Riley RD, Lambert PC, Abo-Zaid G. Meta-analysis of individual participant data: Rationale, conduct, and reporting. BMJ. 2010;340: c221. doi:10.1136/bmj.c221

19. Rijal S, Ostyn B, Uranw S, Rai K, Bhattarai NR, Dorlo TPC, et al. Increasing failure of miltefosine in the treatment of kala-azar in nepal and the potential role of parasite drug resistance, reinfection, or noncompliance. Clin Infect Dis. 2013;56: 1530–1538. doi:10.1093/cid/cit102

20. Sundar S, Singh A, Rai M, Chakravarty J. Single-dose indigenous liposomal amphotericin B in the treatment of Indian visceral leishmaniasis: A phase 2 study. Am J Trop Med Hyg. 2015;92: 513–517. doi:10.4269/ajtmh.14-0259

21. Singh-Phulgenda S, Rashan S, Harriss E, Prabin D, Naylor C, Brack M, et al. Infectious Diseases Data Observatory (IDDO) Visceral Leishmaniasis Library of Clinical Therapeutic Studies : A Protocol for a Living Systematic Review of Clinical Studies. In: Research Square PREPRINT (Version 1). 2022. doi:https://doi.org/10.21203/rs.3.rs-1159661/v1 License:

22. Infectious Diseases Data Observatory. Visceral leishmaniasis Data Inventory. In: iddo.org [Internet]. 2023 [cited 27 Feb 2023]. Available: https://www.iddo.org/document/visceral-leishmaniasis-data-inventory

23. Infectious Diseases Data Observatory. New global collaboration developing standards for standardised data collection across the VL research community. 2020 [cited 28

For peer review only - http://bmjopen.bmj.com/site/about/guidelines.xhtml

Feb 2023]. Available: https://www.iddo.org/news/new-global-collaboration-developing-standards-standardised-data-collection-across-vl-research

24. Chulay JD, Bryceson ADM. Quantitation of amastigotes of Leishmania donovani in smears of splenic aspirates from patients with visceral leishmaniasis. Am J Trop Med Hyg. 1983;32: 475–479. doi:10.4269/ajtmh.1983.32.475

25. Srivastava P, Dayama A, Mehrotra S, Sundar S. Diagnosis of visceral leishmaniasis. Trans R Soc Trop Med Hyg. 2011;105: 1–6. doi:10.1016/j.trstmh.2010.09.006

26. Sudarshan M, Weirather JL, Wilson ME, Sundar S. Study of parasite kinetics with antileishmanial drugs using real-time quantitative PCR in Indian visceral leishmaniasis. J Antimicrob Chemother. 2011;66: 1751–1755. doi:10.1093/jac/dkr185

27. Sundar S, Singh OP. Molecular Diagnosis of Visceral Leishmaniasis. Mol Diagnosis Ther. 2018;22: 443–457. doi:10.1007/s40291-018-0343-y

28. Dorlo TPC, Rijal S, Ostyn B, Vries PJ De, Singh R, Bhattarai N, et al. Failure of Miltefosine in Visceral Leishmaniasis Is Associated With Low Drug Exposure. J Infect Dis. 2014;210: 146–153. doi:10.1093/infdis/jiu039

29. Burke DL, Ensor J, Riley RD. Meta-analysis using individual participant data: one-stage and two-stage approaches, and why they may differ. Stat Med. 2017;36: 855–875. doi:10.1002/sim.7141

30. Riley RD, Moons KGM, Debray TPA. IPD Meta-Analysis for Prognostic Factor Research. In: Richard D. Riley, Jayne F. Tierney LAS, editor. Individual Participant Data Meta-Analysis: A Handbook for Healthcare Research. John Wiley & Sons Ltd; 2021. pp. 421–445. doi:10.1002/9781119333784.ch16

31. Heinze G, Wallisch C, Dunkler D. Variable selection – A review and recommendations for the practicing statistician. Biometrical J. 2018;60: 431–449.

For peer review only

For peer review only - http://bmjopen.bmj.com/site/about/guidelines.xhtml

doi:10.1002/bimj.201700067
32. Sauerbrei W, Royston P, Binder H. Selection of important variables and determination of functional form for continuous predictors in multivariable model building. Stat Med. 2007;26: 5512–5528. doi:10.1002/sim.3148
33. White IR, Royston P, Wood AM. Multiple imputation using chained equations: Issues and guidance for practice. Stat Med. 2011;30: 377–399. doi:10.1002/sim.4067
34. Singh-Phulgenda S, Dahal P, Ngu R, Maguire BJ, Hawryszkiewycz A, Rashan S, et al. Serious adverse events following treatment of visceral leishmaniasis: A systematic review and meta-analysis. PLoS Negl Trop Dis. 2021;15: e0009302. doi:10.1371/journal.pntd.0009302
35. World Health Organization. WHO guidelines for the treatment of visceral leishmaniasis in HIV co-infected patients in East Africa and South-East Asia. World Health OrganizationWorld Health Organization. Geneva, Switzerland; 2022.
36. Riley RD, Debray TPA, Fisher D, Hattle M, Marlin N, Hoogland J, et al. Individual participant data meta-analysis to examine interactions between treatment effect and participant-level covariates: Statistical recommendations for conduct and planning. Stat Med. 2020;39: 2115–2137. doi:10.1002/sim.8516
37. R Core Team. R: A language and environment for statistical computing. Vienna, Austria; 2021. Available: https://www.r-project.org/.
38. StataCorp. Stata Statistical Software: Release 17. College Station, TX; 2021.
39. Sterne JAC, Savović J, Page MJ, Elbers RG, Blencowe NS, Boutron I, et al. RoB 2: A revised tool for assessing risk of bias in randomised trials. BMJ. 2019;366: 1–8. doi:10.1136/bmj.l4898
40. Sterne JA, Hernán MA, Reeves BC, Savović J, Berkman ND, Viswanathan M, et al.

For peer review only - http://bmjopen.bmj.com/site/about/guidelines.xhtml

ROBINS-I: A tool for assessing risk of bias in non-randomised studies of interventions. BMJ. 2016;355: 4–10. doi:10.1136/bmj.i4919

41. Stewart LA, Clarke M, Rovers M, Riley RD, Simmonds M, Stewart G, et al. Preferred reporting items for a systematic review and meta-analysis of individual participant data: The PRISMA-IPD statement. JAMA - J Am Med Assoc. 2015;313: 1657–1665. doi:10.1001/jama.2015.3656

42. Infectious Diseases Data Observatory. Visceral leishmaniasis research agenda. In: 2019 [Internet]. [cited 28 Feb 2023]. Available: https://www.iddo.org/vl/research/research-agenda

43. Rijal S, Sundar S, Mondal D, Das P, Alvar J, Boelaert M. Eliminating visceral leishmaniasis in South Asia: The road ahead. BMJ. 2019;364: k5224.

44. World Health Organization. Regional Strategic Framework for Elimination of Kala-azar from the South-East Asia Region: 2022-2026. New Delhi; 2022.

45. Naylor-leyland G, Collin SM, Gatluak F, Id MDB, Alves F, Mullahzada AW, et al. The increasing incidence of visceral leishmaniasis relapse in South Sudan: A retrospective analysis of field patient data from 2001 – 2018. PLoS Negl Trop Dis. 2022; 1–14. doi:10.1371/journal.pntd.0010696

46. Burza S, Mahajan R, Sanz MG, Sunyoto T, Kumar R, Mitra G, et al. HIV and Visceral Leishmaniasis Coinfection in Bihar,India : An Underrecognized and Underdiagnosed Threat Against Elimination. Clin Infect Dis. 2014;59: 552–555. doi:10.1093/cid/ciu333

47. Alvar J, Aparicio P, Aseffa A, Den Boer M, Cañavate C, Dedet JP, et al. The relationship between leishmaniasis and AIDS: The second 10 years. Clin Microbiol Rev. 2008;21: 334–359. doi:10.1128/CMR.00061-07

48. Cloots K, Marino P, Burza S, Gill N, Boelaert M, Hasker E. Visceral Leishmaniasis-HIV

For peer review only - http://bmjopen.bmj.com/site/about/guidelines.xhtml

Coinfection as a Predictor of Increased Leishmania Transmission at the Village Level in Bihar, India. Front Cell Infect Microbiol. 2021;11: 1–7. doi:10.3389/fcimb.2021.604117

49. Laffitte MN, Leprohon P, Papadopoulou B, Ouellette M. Plasticity of the Leishmania genome leading to gene copy number variations and drug resistance [version 1; peer review :5 approved]. F1000Research. 2022;5: 1–11. doi:https://doi.org/10.12688/f1000research.9218.1

50. Wijnant G, Dumetz F, Dirkx L, Bulte D, Cuypers B, Bocxlaer K Van, et al. Tackling Drug Resistance and Other Causes of Treatment Failure in Leishmaniasis. Front Trop Dis. 2022;3: 1–23. doi:https://doi.org/10.3389/fitd.2022.837460

For peer review only - http://bmjopen.bmj.com/site/about/guidelines.xhtml

Example of Search Strategies: The dates will be different for future searches

Pubmed

((((((((randomized controlled trial[pt] OR controlled clinical trial[pt] OR randomized[tiab] OR placebo[tiab] OR clinical trials as topic[mesh:noexp] OR randomly[tiab] OR trial[ti] NOT (animals[mh] NOT humans [mh]))) AND Clinical Trial[ptyp])) AND (((("Leishmaniasis, Visceral"[Mesh]) OR visceral leishmaniasis[Title/Abstract]) OR kala azar[Title/Abstract]) OR black fever[Title/Abstract]))) OR ((((((((((((((("Pentamidine"[Mesh]) OR "liposomal amphotericin B" [Supplementary Concept]) OR "Amphotericin B"[Mesh]) OR "Paromomycin"[Mesh]) OR "miltefosine" [Supplementary Concept]) OR "Sodium"[Mesh]) OR "8-aminoquinoline" [Supplementary Concept]) OR "Ketoconazole"[Mesh]) OR "Azoles"[Mesh]) OR "Allopurinol"[Mesh]) OR ("Atovaquone"[Mesh] OR "atovaquone, proguanil drug combination" [Supplementary Concept]))) OR ((pentamidine[Title/Abstract] OR ambisome[Title/Abstract] OR amphotericin[Title/Abstract] OR paromomycin[Title/Abstract] OR miltefosine[Title/Abstract] OR pentavalent[Title/Abstract] OR sodium[Title/Abstract] OR sitamaquine[Title/Abstract] OR azole*[Title/Abstract] OR allopurinol[Title/Abstract] OR atovaquone[Title/Abstract] OR ketoconazole[Title/Abstract] OR fluconazole[Title/Abstract] OR metronidazole[Title/Abstract])))) AND (((("Leishmaniasis, Visceral"[Mesh]) OR visceral leishmaniasis[Title/Abstract]) OR kala azar[Title/Abstract]) OR black fever[Title/Abstract])))) NOT (((animals not humans)))
Filters activated: Publication date from 2016/01/01 to 2020/12/31.

- This search strategy uses the PubMed RCT filter for sensitivity- and precision-maximising version (2008). https://work.cochrane.org/pubmed

Database: Embase 1974 to present

Search Strategy:
----
1 exp visceral leishmaniasis/ (9675)
2 "black fever".ti,ab. (17)
3 "kala azar".ti,ab. (2227)
4 "visceral leishmaniasis".ti,ab. (9360)
5 1 or 2 or 3 or 4 (12383)
6 exp randomized controlled trial/ (603493)
7 Controlled clinical study/ (464274)
8 Random$.ti,ab. (1531396)
9 randomization/ (86779)
10 intermethod comparison/ (260073)
11 placebo.ti,ab. (305154)
12 (compare or compared or comparison).ti. (507638)
13 ((evaluated or evaluate or evaluating or assessed or assess) and (compare or compared or comparing or comparison)).ab. (2100417)
14 (open adj label).ti,ab. (78868)
15 ((double or single or doubly or singly) adj (blind or blinded or blindly)).ti,ab. (231216)
16 double blind procedure/ (172220)
17 parallel group$1.ti,ab. (25399)
18 (crossover or cross over).ti,ab. (104779)

For peer review only

19 ((assign$ or match or matched or allocation) adj5 (alternate or group$1 or intervention$1 or patient$1 or subject$1 or participant$1)).ti,ab. (328145)
20 (assigned or allocated).ti,ab. (386517)
21 (controlled adj7 (study or design or trial)).ti,ab. (346624)
22 (volunteer or volunteers).ti,ab. (245818)
23 trial.ti. (298535)
24 6 or 7 or 8 or 9 or 10 or 11 or 12 or 13 or 14 or 15 or 16 or 17 or 18 or 19 or 20 or 21 or 22 or 23 (4650673)
25 5 and 24 (1506)
26 (pentamidine or ambisome or amphotericin or paromomycin or miltefosine or pentavalent or sodium or sitamaquine or azole* or allopurinol or atovaquone or ketoconazole or fluconazole or metronidazole).mp. (1012481)
27 5 and 26 (3701)
28 25 or 27 (4786)
29 (exp animal/ or animal.hw. or nonhuman/) not (exp human/ or human cell/ or (human or humans).ti.) (6396469)
30 28 not 29 (3730)
31 30 (3730)
32 limit 31 to yr="2016 -Current" (774)

- This search strategy uses the Cochrane RCT filter for Embase. https://www.cochranelibrary.com/central/central-creation

Scopus

((TITLE-ABS-KEY ("visceral leishmaniasis" OR "kala azar" OR "black fever")) AND ((TITLE-ABS-KEY (random* OR rct OR placebo OR allocat* OR crossover* OR "cross over" OR trial OR (doubl* W/1 blind*) OR (singl* W/1 blind*))) OR (TITLE-ABS-KEY (control* W/1 trial*)) OR (TITLE-ABS-KEY (cohort*)))) OR ((TITLE-ABS-KEY ("visceral leishmaniasis" OR "kala azar" OR "black fever")) AND (TITLE-ABS-KEY (pentamidine OR ambisome OR amphotericin OR paromomycin OR miltefosine OR pentavalent OR sodium OR sitamaquine OR azole* OR allopurinol OR atovaquone OR ketoconazole OR fluconazole OR metronidazole))) AND (EXCLUDE (EXACTKEYWORD , "Animals") OR EXCLUDE (EXACTKEYWORD , "Animal") OR EXCLUDE (EXACTKEYWORD , "Animal Experiment") OR EXCLUDE (EXACTKEYWORD , "Mouse") OR EXCLUDE (EXACTKEYWORD , "Mice") OR EXCLUDE (EXACTKEYWORD , "Dogs")) AND (LIMIT-TO (LIMIT-TO (PUBYEAR , 2020) OR (PUBYEAR , 2019) OR LIMIT-TO (PUBYEAR , 2018) OR LIMIT-TO (PUBYEAR , 2017) OR LIMIT-TO (PUBYEAR , 2016))

Web of Science Core Collection

1. TOPIC: ("visceral leishmaniasis" OR "kala azar" OR "black fever")
2. TOPIC: (random* OR rct OR placebo OR allocat* OR crossover* OR "cross over" OR trial OR (doubl* near/1 blind*) OR (singl* near/1 blind*))
3. TOPIC: (control* near/1 trial*)
4. TOPIC: (cohort*)
5. #4 OR #3 OR #2
6. #5 AND #1
7. TOPIC: (pentamidine OR ambisome OR amphotericin OR paromomycin OR miltefosine OR pentavalent OR sodium OR sitamaquine OR azole* OR allopurinol OR atovaquone OR ketoconazole OR fluconazole OR metronidazole)

For peer review only

8. #7 AND #1
9. #8 OR #6
10. #8 OR #6
11. Refined by: PUBLICATION YEARS: (2020 OR 2019 OR 2018 OR 2017 OR 2016)

Cochrane Central Register of Controlled Trials
Issue 5 of 12, May 2020

#1 MeSH descriptor: [Leishmaniasis, Visceral] explode all trees 39
#2 "black fever" 1
#3 "kala azar" 119
#4 "visceral leishmaniasis" 242
#5 #1 or #2 or #3 or #4 283
Custom Range: 2016 to 2020

World Health Organization Global Index Medicus https://www.globalindexmedicus.net/

tw:(tw:(("visceral leishmaniasis" OR "kala azar" OR "black fever")) AND (instance:"ghl") AND (year_cluster:("2016" OR "2017" OR "2018" OR "2019" OR “2020”))) AND (instance:"ghl")

ClinicalTrials.gov Advanced Search https://clinicaltrials.gov/ct2/search/advanced?

Condition or disease: visceral leishmaniasis OR kala azar OR black fever

WHO International Clinical Trials Registry Platform http://apps.who.int/trialsearch/

visceral leishmaniasis OR kala azar OR black fever – Trials at ALL stages
2016 – date

NOTE: THIS PLATFORM IS UNAVAILABLE ON 14/05/2020

For peer review only

PRISMA-P (preferred reporting items for systematic review and meta-analysis protocols) 2015 checklist: recommended items to address in a systematic review protocol

Section and topic	Item No	Checklist item
**Administrative information**		
Title:		
Identification	1a	A protocol for individual participant data meta-analysis; page 1; line 2-3.
Update	1b	Not applicable
Registration	2	PROSPERO registration: CRD42021284622; page 3; lines 47-48.
Authors:		
Contact	3a	Name with institutional affiliations of all authors and e-mail address corresponding authors; page 1; line 5-20.<br>E-mail address of all authors; page 3; line 50-66
Contributions	3b	Authors' contribution; page 23; line 453-456.
Amendments	4	Not applicable
Support:		
Sources	5a	Funding; page 23; line 458-461.
Sponsor	5b	Funding; page 23; line 458-461.
Role of sponsor or funder	5c	Funding agency's role; page 23; line 461.
**Introduction**		
Rationale	6	Rationale; page 5-6; line 94-118.
Objectives	7	Study objectives; page 6; line 120-131.
**Methods**		
Eligibility criteria	8	PICOT statement, study eligibility and patient eligibility; page 7-8
Information sources	9	Identification of relevant studies; page 10; line 202-214.
Search strategy	10	Identification of relevant studies; page 10; line 202-214.
Study records:		
Data management	11a	Data management; page 11; line 222-230.

Section and topic	Item No	Checklist item
Selection process	11b	Identification of relevant studies; page 10; line 202-214.
Data collection process	11c	Collation of IPD; page 11; line 216-221.
Data items	12	Statistical methods for primary and secondary outcomes; page 13-14
Outcomes and prioritization	13	Outcomes; page 8-10; line 159-200.
Risk of bias in individual studies	14	Risk of bias assessment in included studies and in the IPD-MA; page 17-18; line 357-375.
Data synthesis	15a	Missing data; page 15; line 316-325.
	15b	Assessment of risk of potential bias in missing studies; page 18; line 369-375.
	15c	Subgroup analyses, Sensitivity analyses; page 17; line 320-339.
	15d	Assessment of risk of potential bias in missing studies; page 16; line 326-340.
Meta-bias(es)	16	Risk of bias assessment in included studies and in the IPD-MA; page 17-18; line 357-375.
Confidence in cumulative evidence	17	Risk of bias assessment in included studies and in the IPD-MA; page 17-18; line 357-375.

For peer review only
